# Well-defined aqueous nanoassemblies from amphiphilic *meta*-terphenyls and their guest incorporation†
†Electronic supplementary information (ESI) available: Experimental procedures and physical data. See DOI: 10.1039/c5sc01545f



**DOI:** 10.1039/c5sc01545f

**Published:** 2015-06-09

**Authors:** Yusuke Okazawa, Kei Kondo, Munetaka Akita, Michito Yoshizawa

**Affiliations:** a Chemical Resources Laboratory , Tokyo Institute of Technology , 4259 Nagatsuta, Midori-ku , Yokohama 226-8503 , Japan . Email: yoshizawa.m.ac@m.titech.ac.jp

## Abstract

Spherical molecular assemblies with diameters of ∼2 nm were quantitatively formed in water from new amphiphilic *meta*-terphenyls with two hydrophilic pendants at the central benzene ring. Whereas intermolecular interactions between small aromatic rings are typically weak, the obtained nanoassemblies are stable enough at wide-ranging concentrations and mostly remain intact even in the presence of similar nanoassemblies with polyaromatic frameworks. The nanoassembly with pentamethyl-substituted terminal benzene rings provides superior host capability for fluorescent dyes in water.

## Abstract 2

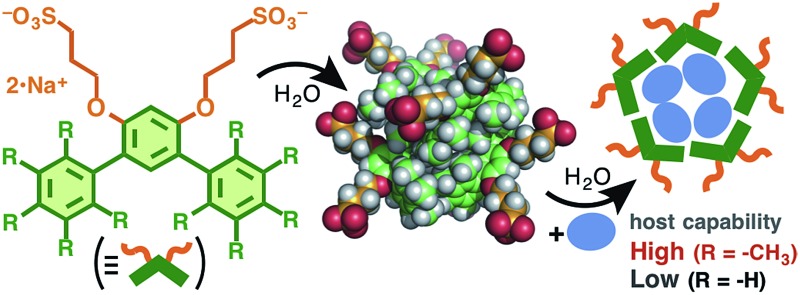
Spherical molecular assemblies with diameters of ∼2 nm were quantitatively formed in water from new amphiphilic *meta*-terphenyls and the nanoassembly with methyl groups provides superior host capability for fluorescent dyes.

## Introduction

A benzene ring, the minimal unit of polyaromatic hydrocarbons, is widely utilized for the construction of various molecular architectures.^1,2^ However, intermolecular interactions between such small aromatic rings are relatively weak as compared with those between extended polyaromatic rings (*e.g.*, anthracene and pyrene) therefore the certain formation of well-defined, benzene-based molecular assemblies requires core frameworks containing multiple benzene rings linked covalently.^3,4^ For instance, Lee *et al.* reported that linear amphiphilic compounds with a quaterphenyl framework (C_6_H_5_–C_6_H_4_–C_6_H_4_–C_6_H_5_) generate large micellar or vesicle-like assemblies (>∼20 nm in diameter) in water.^5^ The bent oligophenyl (C_6_H_5_–(C_6_H_4_)_7_–C_6_H_5_) amphiphiles also form huge tubular assemblies.^5^ On the other hand, small frameworks composed of two or three benzene rings have not proven useful for the subunits of discrete molecular assemblies.^6^

**Fig. 1:**
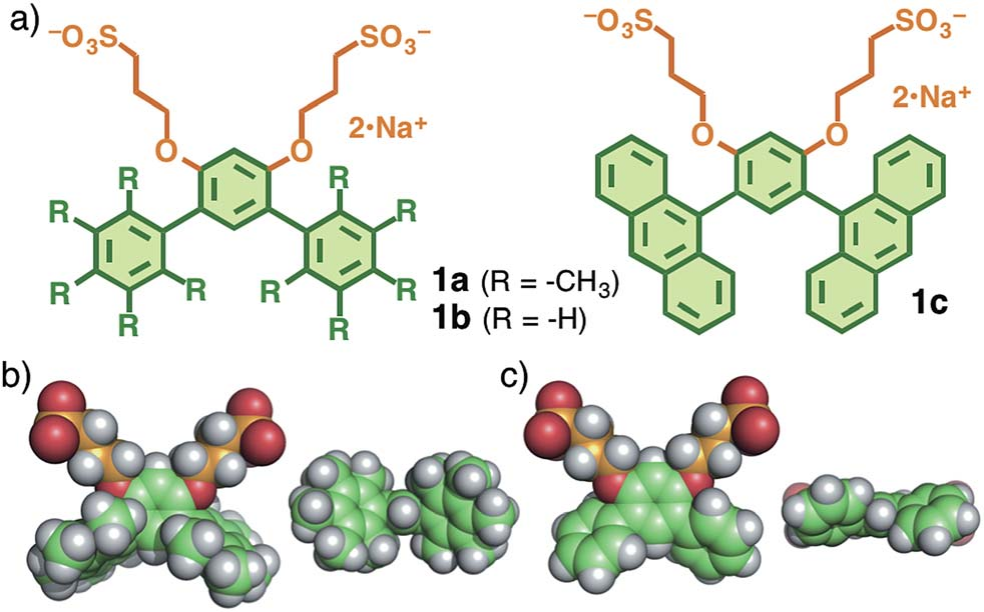
(a) V-shaped amphiphilic compounds **1a–c**. The optimized structures of (b) **1a** and (c) **1b** (side and front views) without the counterions, obtained by DFT calculations (B3LYP/6-31G(d,p) level).

Here we employed a *meta*-terphenyl framework to explore new molecular assemblies with well-controlled size and shape distributions as well as guest incorporation ability. V-shaped amphiphilic compounds **1a** and **1b**, designed here (Fig. 1a), are composed of a *meta*-terphenyl skeleton with two hydrophilic pendants at the central benzene ring. To force the terminal benzene rings to adopt an orthogonal conformation with respect to the central ring, methyl groups were employed as substituents on the terminal rings (**1a**; Fig. 1b).^7^ In this context, we have recently revealed that a similar amphiphilic molecule **1c** with a bent *meta*-di(anthryl)benzene framework generates a spherical polyaromatic assembly **2c** in water through effective π-stacking interactions.^8^ Herein, we report the quantitative formation of well-defined, spherical assemblies **2a** and **2b** from ∼5 molecules of **1a** and **1b** in aqueous media. The pentamethylbenzene-shelled assembly **2a** effectively incorporates well-known fluorescent dyes (*i.e.*, fluorescein and Eosin Y) in water at room temperature and the emissive colors of the encapsulated dyes are altered by the host–guest interactions.

## Results and discussion

### Quantitative formation of the spherical nanoassemblies

**Fig. 2:**
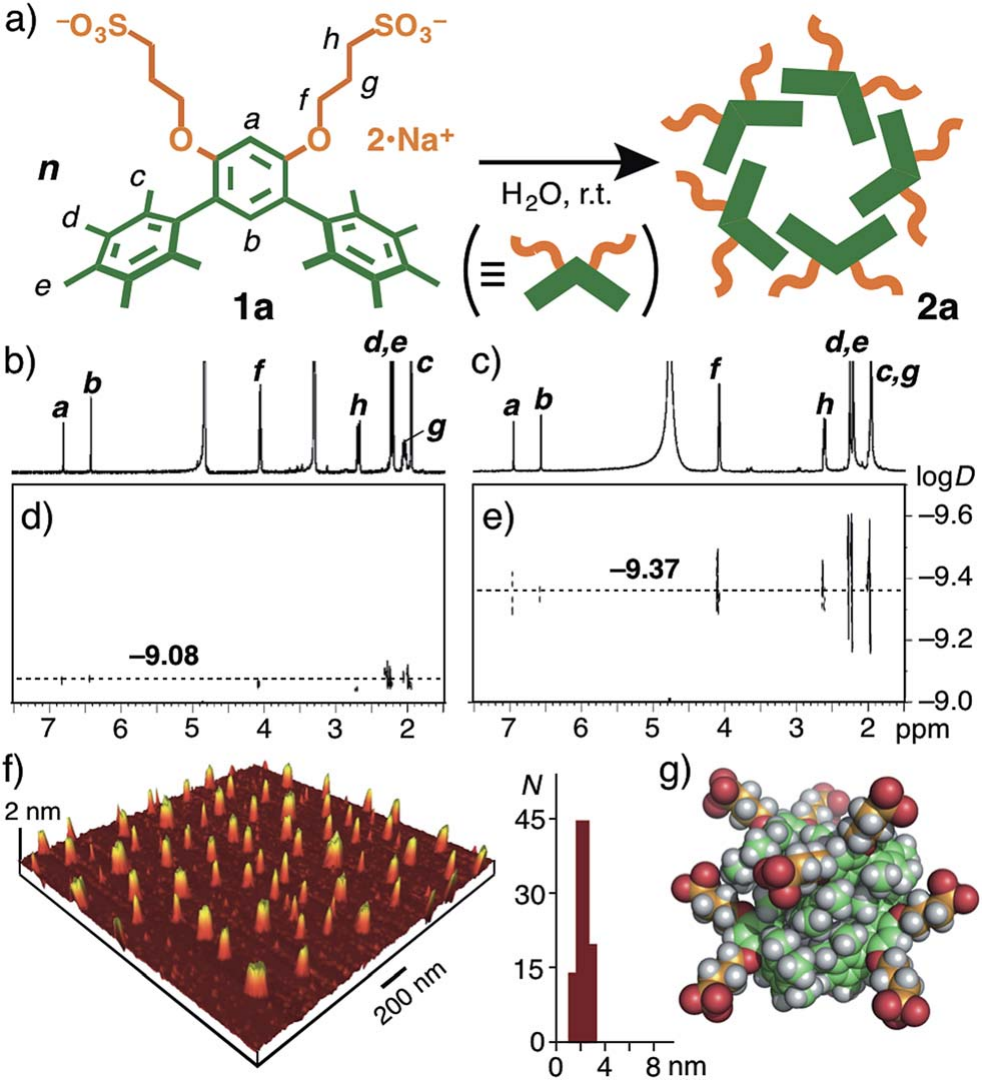
(a) Schematic representation of the formation of spherical nanoassembly **2a** from V-shaped amphiphilic compound **1a** in water. ^1^H NMR and DOSY spectra (400 MHz, 25 °C) of (b and d) **1a** in CD_3_OD and (c and e) **2a** in D_2_O (2.0 mM based on **1a**). (f) AFM image of **2a** on mica and the size and number (*N*) distribution. (g) Molecular modeling of **2a**, composed of five molecules of **1a**.

V-shaped amphiphilic compound **1a** was synthesized through four step reactions with ∼35% overall yield.^9^ Once a white solid of **1a** (2.0 μmol) was dissolved in water (1.0 mL) at room temperature, spherical assembly **2a** was quantitatively formed within 1 min (Fig. 2a). The ^1^H NMR spectrum of **2a** in D_2_O is similar to that of **1a** in CD_3_OD and contains two aromatic (*H*_a,b_) and six aliphatic (*H*_c–h_) signals (Fig. 2b and c). On the other hand, the observed diffusion coefficients (*D*) of **2a** in D_2_O and **1a** in CD_3_OD were evidently different. The diffusion-ordered spectroscopy (DOSY) NMR spectrum of **2a** displayed a single band at a *D* of 4.3 × 10^–10^ m^2^ s^–1^ (Fig. 2e), which is smaller than that of **1a** (8.3 × 10^–10^ m^2^ s^–1^; Fig. 2d). On the basis of the *D* value and the Stokes–Einstein equation, the core diameter of **2a** was calculated to be about 1 nm. Further structural evidence for **2a** was obtained by atomic force microscopy (AFM). We observed uniform, spherical particles with an average height of 2.2 nm on the AFM image (Fig. 2f), prepared by drop casting from an aqueous solution of **2a** (1.0 mM based on **1a**) on a mica surface. The diameter of the spherical assembly of pentamer (**1a**)_5_ including the outer sulfonate groups (2.5 nm)^10^ is comparable to that of **2a** obtained by the AFM analysis (Fig. 2g).

It is worth noting that the methyl groups on the terminal benzene rings of **1a** are not essential for the formation of well-defined, small nanoassemblies. Thus, in a manner similar to **2a**, simple V-shaped amphiphile **1b** without the methyl groups^7^ quantitatively formed spherical assembly **2b** in water at room temperature. The size of the product is slightly smaller than that of **2a** but the shape is quite similar to that of **2a**, as confirmed by DOSY NMR, DLS, and AFM analyses (Fig. S38–41†).^9^ In agreement with our previous study of amphiphile **1c** with a bent polyaromatic framework,^8^ the *meta*-terphenyl moiety was therefore proved to be one of the key skeletons in this supramolecular system.

### Photophysical properties and stability of the spherical nanoassemblies

Assemblies **2a** and **2b** turned out to be stable enough in water under ambient conditions. Dynamic light scattering (DLS) analysis of **2a** indicated the exclusive existence of small particles (*d*_av._ = 1.6 nm) with a sharp size distribution (±1 nm) at the monomer concentration of 1.0 mM. Concentration-dependent DLS studies revealed that the particles of **2a** and **2b** remained intact at up to 10 mM in water at room temperature for more than one week (Fig. S39 and 40†). The water-solubility of **2a** and **2b** is higher than that of **2c** (up to ∼2 mM based on **1c**). Concentration-dependent fluorescence studies (in the range of 0.001–1.0 mM based on **1a**) elucidated that the critical micelle concentration (CMC) value of **2a** is ∼0.005 mM.

**Fig. 3:**
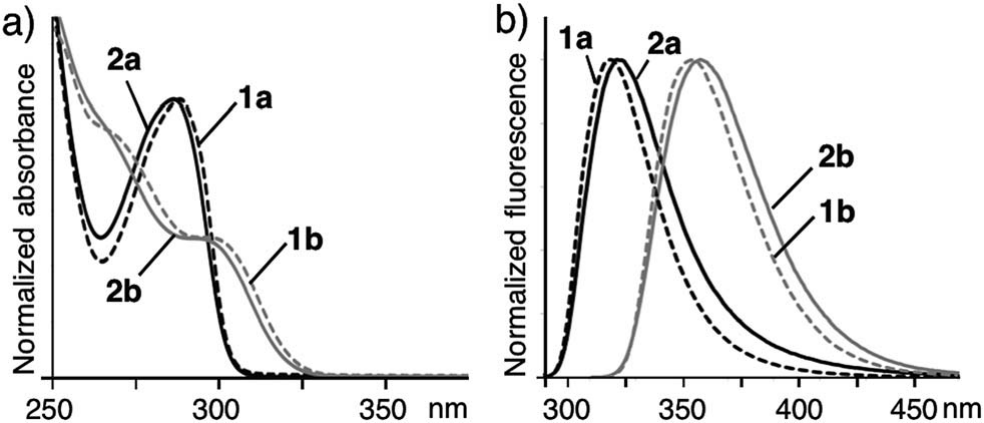
(a) Normalized UV-visible and (b) fluorescence spectra (r.t.) of nanoassemblies **2a** and **2b** in H_2_O (1.0 mM based on **1a** and **1b**) and monomers **1a** and **1b** in CH_3_OH. Excitation wavelengths: *λ*_ex_ = 286 nm for **1a** and **2a**, *λ*_ex_ = 299 nm for **1b** and **2b**.

The UV-visible spectra of monomer **1a** in methanol and assembly **2a** in water exhibited similar absorption bands at *λ*_max_ = ∼290 nm (Fig. 3a). The absorption bands are blue-shifted (Δ*λ* = ∼10 nm) as compared with those of **1b** and **2b**. Similarly, the emission bands of **1a** and **2a** are also blue-shifted (Δ*λ*_max_ = ∼30 nm) with respect to those of **1b** and **2b** (Fig. 3b). These photophysical properties can be explained by the conformational difference between the two amphiphiles. Sterically hindered **1a** adopts a rigidly bent structure for the methyl-substituted *meta*-terphenyl moiety but **1b** adopts a slightly twisted, planar structure for the *meta*-terphenyl framework with an extended π-conjugated system, as also suggested by the optimized structures of **1a** and **1b** (Fig. 1b and c).

**Fig. 4:**
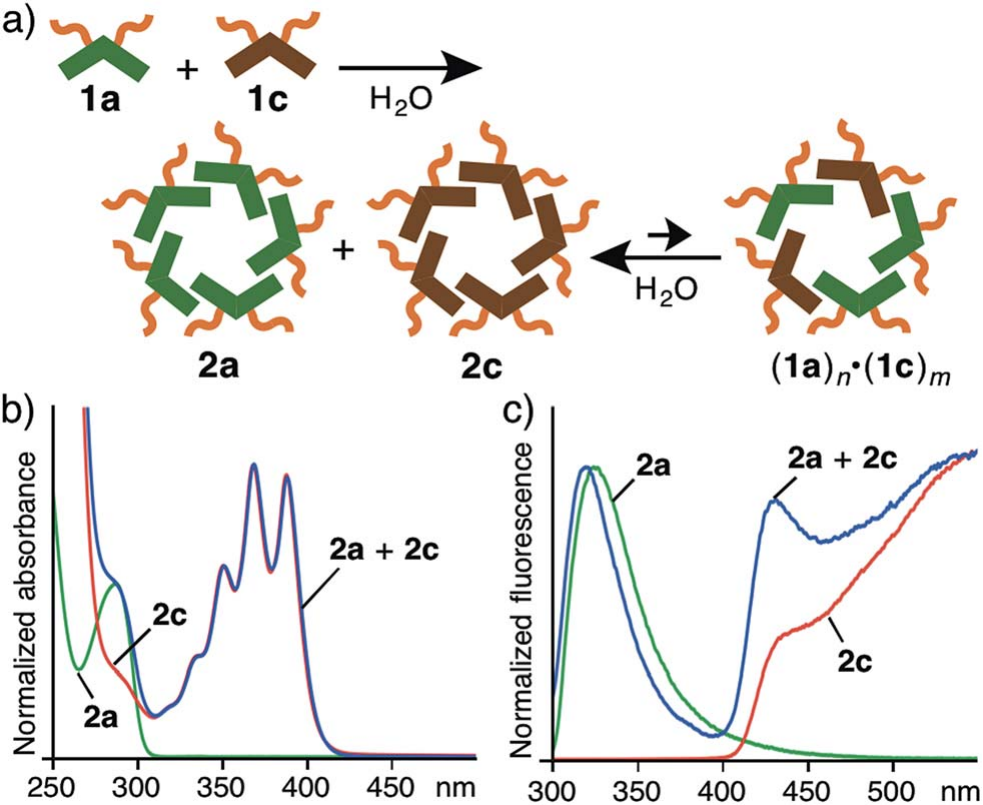
(a) Schematic representation of the selective formation of nanoassemblies **2a** and **2c** from a mixture of **1a** and **1c** in water. (b) Normalized UV-visible and (c) fluorescence spectra (H_2_O, r.t., *λ*_ex_ = 286 nm) of **2a**, **2c**, and **2a** + **2c** (1.0 mM based on **1a** and **1c** each).

Mixing two linear amphiphilic compounds with different hydrophobic alkyl chains usually generates a complex mixture of spherical aggregates with a statistical distribution of the amphiphiles in water.^11^ In contrast, the combination of V-shaped amphiphiles **1a** and **1c** led to an equilibrium mixture of nanoassemblies, mainly including **2a** and **2c** (Fig. 4a). When **1a** and **1c** (in a 1 : 1 ratio) were agitated in H_2_O (1.0 mM) at room temperature for 1 min, the resultant solution was analyzed by UV-visible and fluorescence spectroscopies. The observed absorption and emission bands nearly overlapped with the sum of the bands of assemblies **2a** and **2c** (Fig. 4b and c). No coalesced emission bands were observed, in contrast to similar polyaromatic assemblies (Fig. S47 and 48†).^9^ In addition, the mixed solution of **2a** and **2c** in H_2_O at room temperature (even after heating at 80 °C for 10 min) exhibited the same spectrum. These behaviors are most probably caused by the difference of the major intermolecular interactions between the two amphiphiles: **2a** forms through hydrophobic effects but **2c** forms through π-stacking interactions in aqueous solutions.

### Effective incorporation of fluorescent dyes

**Fig. 5:**
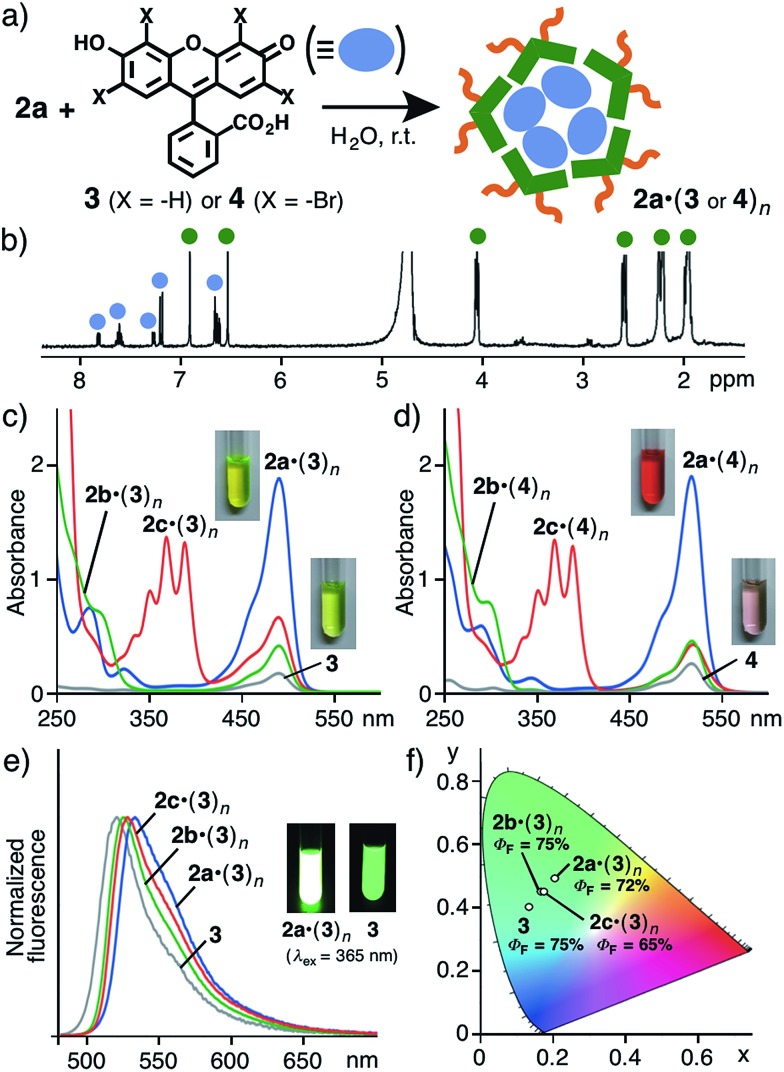
(a) Schematic representation of the incorporation of fluorescein (**3**) or Eosin Y (**4**) by nanoassembly **2a** in water. (b) ^1^H NMR spectrum (400 MHz, r.t.) of **2a·(3)_*n*_** in D_2_O. UV-visible spectra (r.t. H_2_O) of (c) nanoassemblies **2a–c·(3)_*n*_** and (d) **2a–c·(4)_*n*_** (1.0 mM based on **1a–c**). (e) Normalized fluorescence spectra (r.t. H_2_O, *λ*_ex_ = 487 nm) and (f) CIE coordinate diagram and quantum yields of **3** and **2a–c·(3)_*n*_**.

Surprisingly, the guest incorporation ability of **2a** and **2b** is strikingly different in water. Nanoassembly **2a** with its bent bis-pentamethylbenzene frameworks promoted guest incorporation. For example, an excess amount of fluorescein (**3**; 1.0 μmol) with slightly hydrophilic properties was suspended in a D_2_O solution (2.0 mL) of **2a** (2.0 μmol based on **1a**) and the resultant mixture was stirred at room temperature for 1 h (Fig. 5a). The filtration of the suspended mixture yielded **2a·(3)_*n*_** as a clear pale-yellow solution. In the ^1^H NMR spectrum, new signals derived from encapsulated **3** were clearly observed in the range of 6.6–7.9 ppm (Fig. 5b), whereas the proton signals of free **3** were hardly detectable in water (Fig. S50†). The UV-visible spectrum showed a new prominent absorption band at *λ*_max_ = 487 nm assignable to the incorporated guest molecules (Fig. 5c). In stark contrast, similar assemblies **2b** and **2c** exhibited weak binding abilities for **3** under the same conditions, as revealed by the UV-visible analysis (Fig. 5c).

Eosin Y (**4**) was also effectively incorporated into **2a** in water at room temperature to give a red solution due to the formation of **2a·(4)_*n*_** (Fig. 5d).^9,12^ Assemblies **2b** and **2c** showed lower binding abilities for **4**. On the basis of the absorption intensities, the concentrations of **3** and **4** in the obtained aqueous solutions of **2a·(3)_*n*_** and **2a·(4)_*n*_** were estimated to be 0.32 and 0.30 mM, respectively.^13^ The values are >20-times larger than those of the saturated solutions of **3** and **4** in water without **2a–c**. Furthermore, the intense fluorescence band (*Φ*_F_ = 72%) of the incorporated **3** within **2a** was found at *λ*_max_ = 532 nm, which is slightly red-shifted (Δ*λ*_max_ = 13 nm) as compared with that of free **3** due to being enclosed in the pentamethylbenzene frameworks (Fig. 5e). The difference in the total emission colors of **3** and **2a–c·(3)_*n*_** is clearly revealed in the CIE chromaticity diagram (Fig. 5f). Thus, it is revealed that the bent conformation of the pentamethyl-substituted *meta*-terphenyl framework is of importance for the formation of a nanoassembly with an inner space capable of binding guest molecules.

## Conclusions

In conclusion, we have revealed that *meta*-terphenyl, a small benzene oligomer, acts as a useful building block for the quantitative formation of well-defined, spherical assemblies (∼2 nm in diameter) in water by attaching hydrophilic groups to the central benzene ring. The nanoassemblies are stable enough in a wide range of concentrations and remain intact even when mixed with a similar nanoassembly with polyaromatic frameworks. Moreover, pentamethyl substitution on the terminal benzene rings of the amphiphile forces the *meta*-terphenyl framework to adopt a bent conformation, which imparts the nanoassembly with a superior hosting capability for fluorescent dyes in water. The present study demonstrated the utility of a simple C_6_H_5_–C_6_H_4_–C_6_H_5_ skeleton for discrete supramolecular systems with designed structures and functions.
