# Supplementary material for: Well-defined aqueous nanoassemblies from amphiphilic meta-terphenyls and their guest incorporation
Source: Chem Sci. 2015 Jun 9;6(8):5059–62. doi: 10.1039/c5sc01545f (PMC5499508; doi:10.1039/c5sc01545f)
Supplement: Supplementary file 1 [file SC-006-C5SC01545F-s001.pdf]

Electronic Supplementary Material (ESI) for Chemical Science.
This journal is © The Royal Society of Chemistry 2015

Supporting Information

Well-defined Aqueous Nanoassemblies from Amphiphilic *meta*-Terphenyls and Their Guest Incorporation

Yusuke Okazawa, Kei Kondo, Munetaka Akita, and Michito Yoshizawa*

Chemical Resources Laboratory, Tokyo Institute of Technology, 4259 Nagatsuta, Midori-ku, Yokohama 226-8503, Japan; *e-mail: yoshizawa.m.ac@m.titech.ac.jp

Contents

- Materials and methods
- Synthesis of **4a** (^{1}H & ^{13}C-NMR, NOESY, & HSQC spectra)
- Synthesis of **5a** (^{1}H & ^{13}C-NMR, NOESY, & HSQC spectra)
- Synthesis of **1a** (^{1}H & ^{13}C-NMR, HH-COSY, NOESY, HSQC, & HR MS spectra)
- Self-assembly of **2a** (^{1}H & ^{13}C-NMR, HH-COSY, NOESY, HSQC, DOSY spectra)
- Synthesis of **4b** (^{1}H & ^{13}C-NMR, HH-COSY, & HSQC spectra)
- Synthesis of **5b** (^{1}H & ^{13}C-NMR, HH-COSY, & HSQC spectra)
- Synthesis of **1b** (^{1}H & ^{13}C-NMR, HH-COSY, HSQC, & HR MS spectra)
- Self-assembly of **2b** (^{1}H & ^{13}C-NMR, HH-COSY, HSQC, DOSY spectra)
- DLS analysis of **2a** and **2b**
- AFM analysis of **2b**
- Concentration-dependent UV-vis and fluorescence spectra of **2a** and **2b**
- ^{1}H-NMR spectra of **2a**, **2a+2c**, and **2c**
- UV-vis and fluorescence spectra of **2a+2c**
- Synthesis and DLS analysis of **2a-c**⊃(**3**)$_n$ and **2a-c**⊃(**4**)$_n$
- UV-vis and fluorescence spectra of **2c**+**2d** and **2c**+**2e**
- Optimized structures of spherical assemblies (**1a**)$_n$
- ^{1}H-NMR spectra of **2a•**(**3**)$_n$ and **3**.

Materials and methods

NMR: Bruker AVANCE-400 (400 MHz) or ASCEND-500 (500 MHz), MALDI-TOF MS: Shimadzu AXIMA-CFR Plus, ESI-TOF MS: Bruker micrOTOF II, Particle Size Analysis (DLS): Wyatt Technology DynaPro NanoStar, AFM: Asylum Reseach Cypher S, FT IR: JASCO FT/IR-4200, UV-vis: JASCO V-670DS, Fluorescence: HITACHI F7000, Absolute PL quantum yield: Hamamatsu Quantaurus-QY C11347-01, Elemental analysis: LECO CHNS-932 VTF-900.

Solvents and reagents: TCI Co., Ltd., Wako Pure Chemical Industries Ltd., Kanto Chemical Co., Inc., Sigma-Aldrich Co., and Cambridge Isotope Laboratories, Inc.

Compounds: 1,5-dibromo-2,4-dimethoxybenzene and **1c** were synthesized according to previously published procedures.[1,2]

References

[1] E. Kiehlmannan, D. R. W. Lauener, *Can. J. Chem.*, **1989**, *67*, 335–344.

[2] K. Kondo, A. Suzuki, M. Akita, M. Yoshizawa, *Angew. Chem. Int. Ed.*, **2013**, *52*, 2308–2312.

Synthesis of 4a YO-97

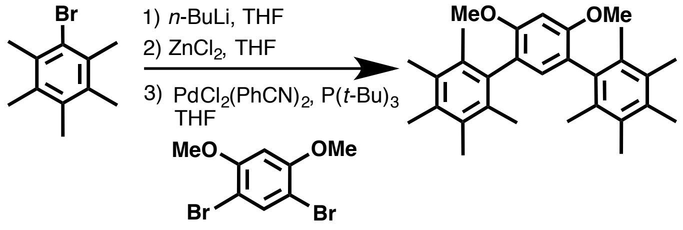

1-Bromopentamethylbenzene (2.000 g, 8.805 mmol) and dry THF (50 mL) were added to a 2-necked 300 mL glass flask filled with N_2. A hexane solution (2.69 M) of *n*-butyllithium (3.6 mL, 9.7 mmol) was added dropwise to the flask at –80 °C under N_2. After stirring at –80 °C for 2 h, a dry THF solution (30 mL) of $ZnCl_2$ (1.560 g, 11.44 mmol) was added to the solution. The resultant mixture was further stirred at –80 °C for 1 h and then the solution was warmed to r.t. for 1 d to obtain pentamethylphenylzinc chloride. 1,5-Dibromo-2,4-dimethoxybenene (0.886 g, 2.99 mmol), $PdCl_2(PhCN)_2$ (80 mg, 0.2 mmol), and dry THF (30 mL) were added to a 50 mL glass flask filled with N_2. A hexane solution (0.95 M) of tri-*tert*-butylphosphine (0.4 mL, 0.4 mmol) was added to the 50 mL flask. After stirring at r.t. for 30 min, the mixture was added to the 300 mL flask and then the resulted solution was further stirred at 85 °C for 2 d. The precipitated crude product was extracted with CH_2Cl_2 and hexane (as an azeotropic solvent). The solution was concentrated under reduced pressure. The crude product was collected and washed with CH_3OH to afford **4a** (0.734 g, 1.70 mmol, 57% yield) as a pale gray solid. ^{1}H NMR (400 MHz, $CDCl_3$, r.t.): δ 2.04 (s, 12H), 2.27 (s, 12H), 2.30 (s, 6H), 3.84 (s, 6H), 6.66 (s, 1H), 6.69 (s, 1H). ^{13}C NMR (100 MHz, $CDCl_3$, r.t.): δ 16.8 (CH_3), 17.0 (CH_3), 18.1 (CH_3), 55.8 (CH_3), 95.6 (CH), 123.5 (C_q), 132.1 (C_q), 132.5 (C_q), 133.8 (C_q), 133.9 (CH), 135.7 (C_q), 156.7 (C_q). FT-IR (KBr, cm^{-1}): 3437, 2990, 2928, 2877, 2727, 1603, 1573, 1502, 1456, 1377, 1321, 1277, 1250, 1198, 1166, 1108, 1065, 1032, 997, 921, 903, 815, 785, 628. MALDI-TOF MS (dithranol): *m/z* Calcd. for $C_{30}H_{38}O_2$: 430.29, Found 430.16 $[M]^+$. E.A.: Calcd. for $C_{30}H_{38}O_2$: C, 83.67; H, 8.89. Found: C, 83.54; H, 9.14.

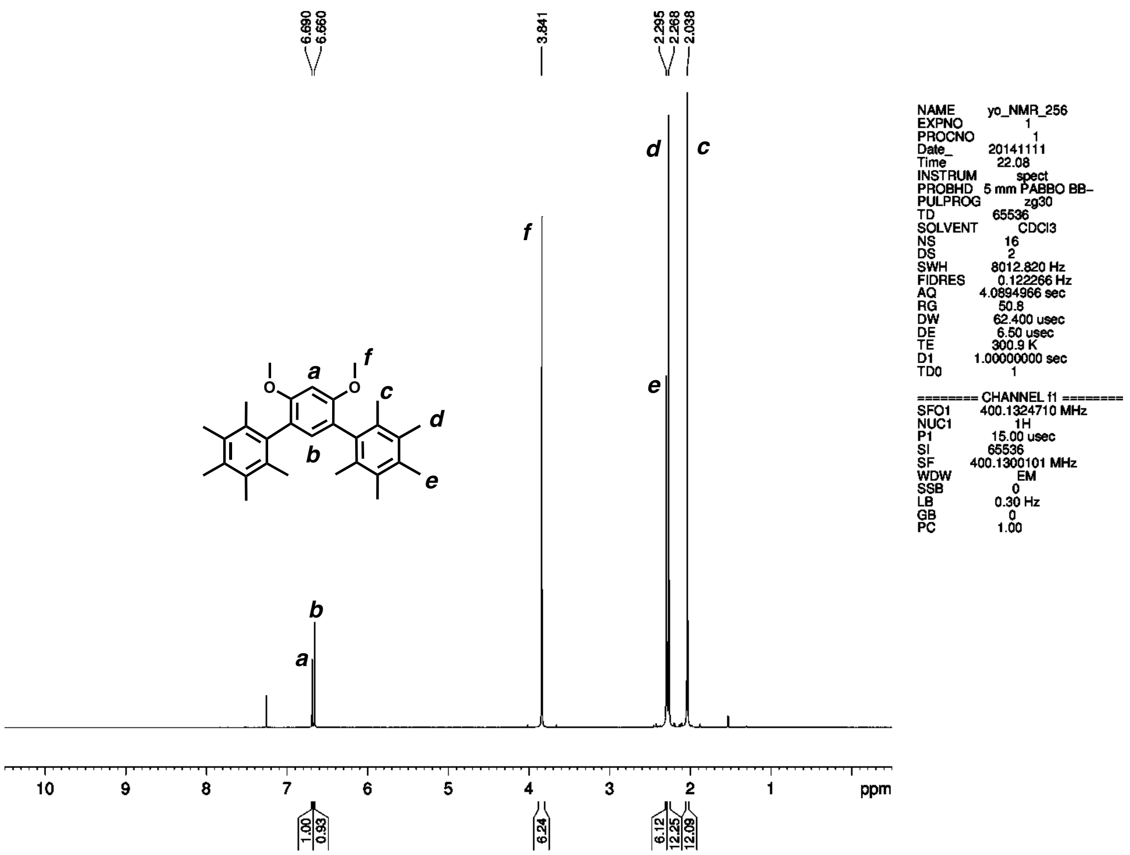

Figure S1. ^{1}H NMR spectrum (400 MHz, $CDCl_3$, r.t.) of **4a**.

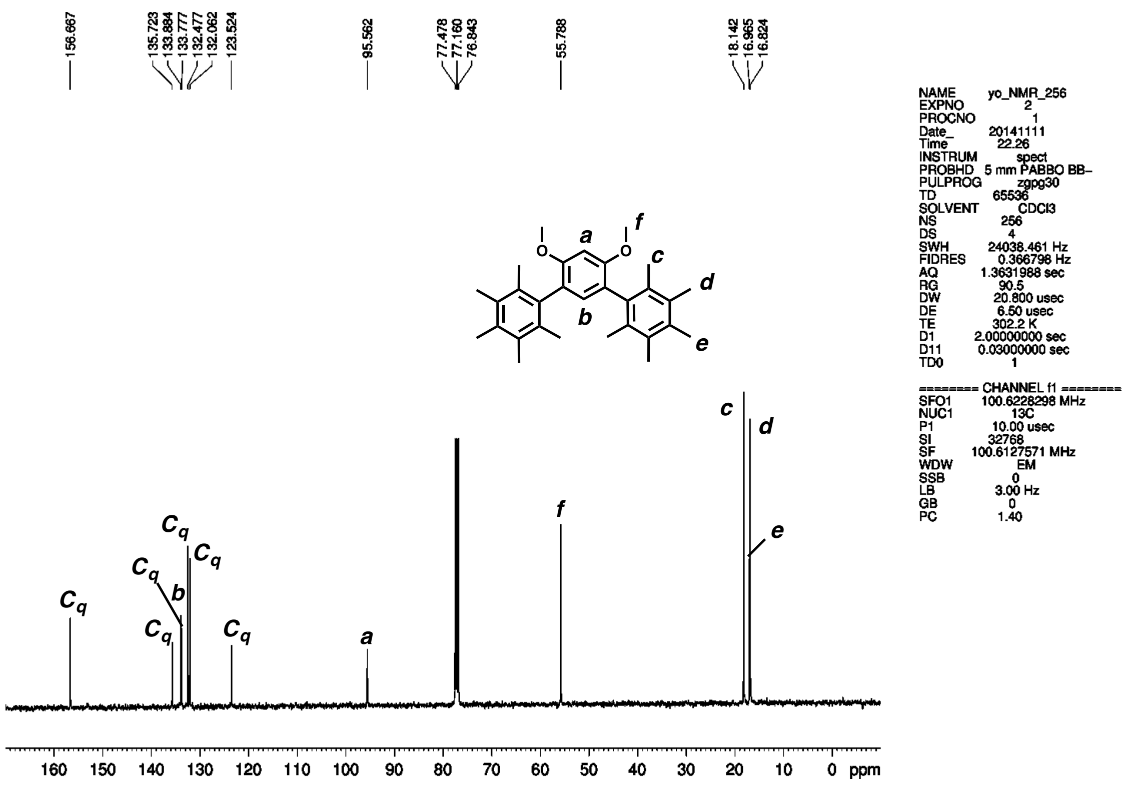

Figure S2. ^{13}C NMR spectrum (100 MHz, $CDCl_3$, r.t.) of **4a**.

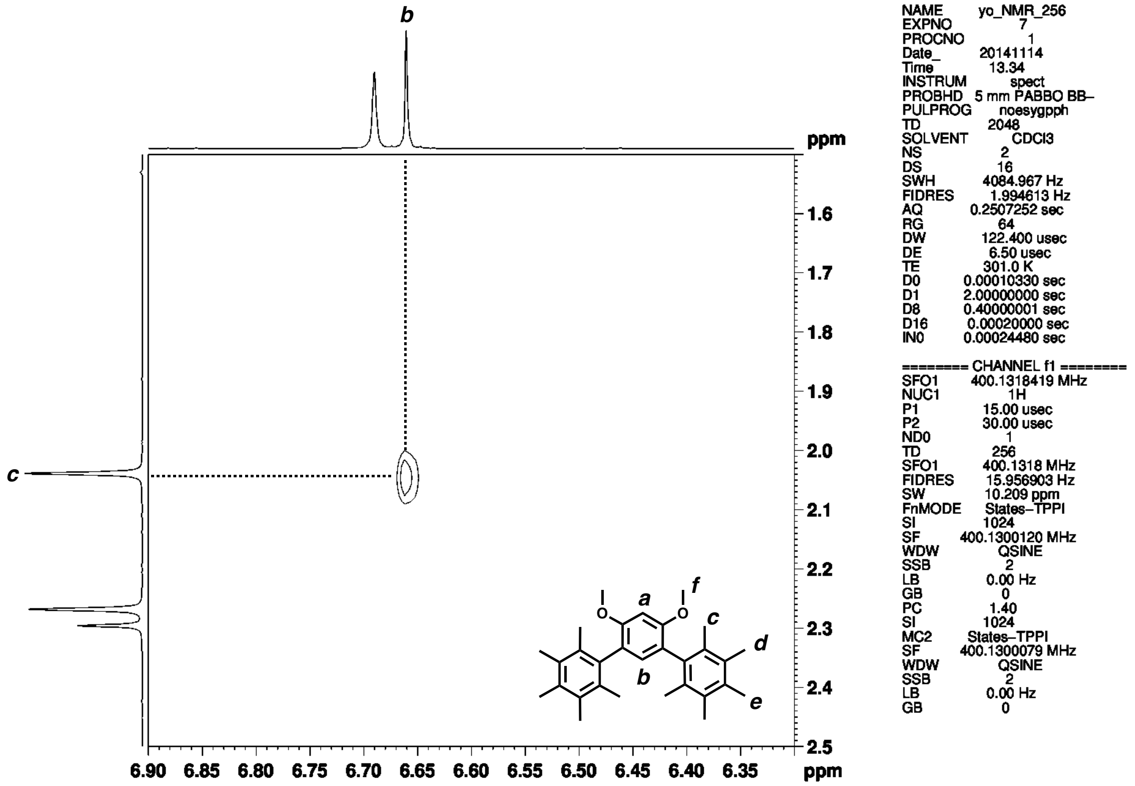

Figure S3a. NOESY spectrum (400 MHz, $CDCl_3$, r.t.) of **4a**.

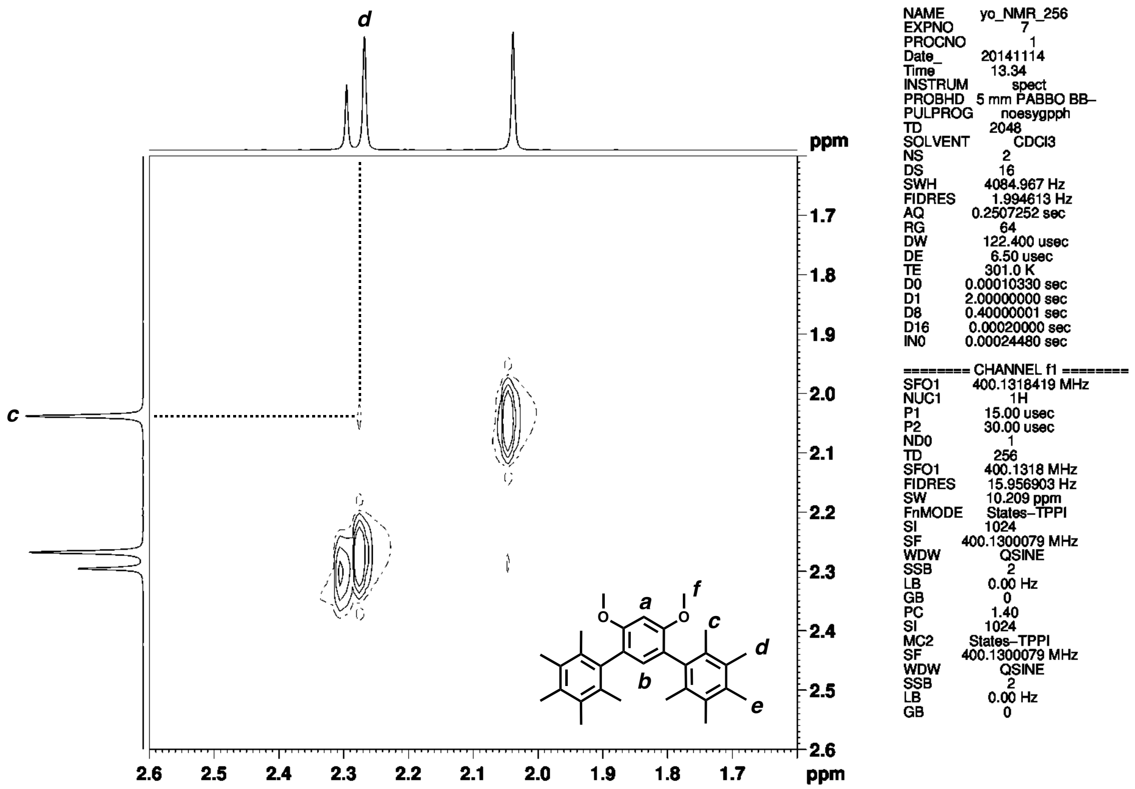

Figure S3b. NOESY spectrum (400 MHz, $CDCl_3$, r.t.) of **4a**.

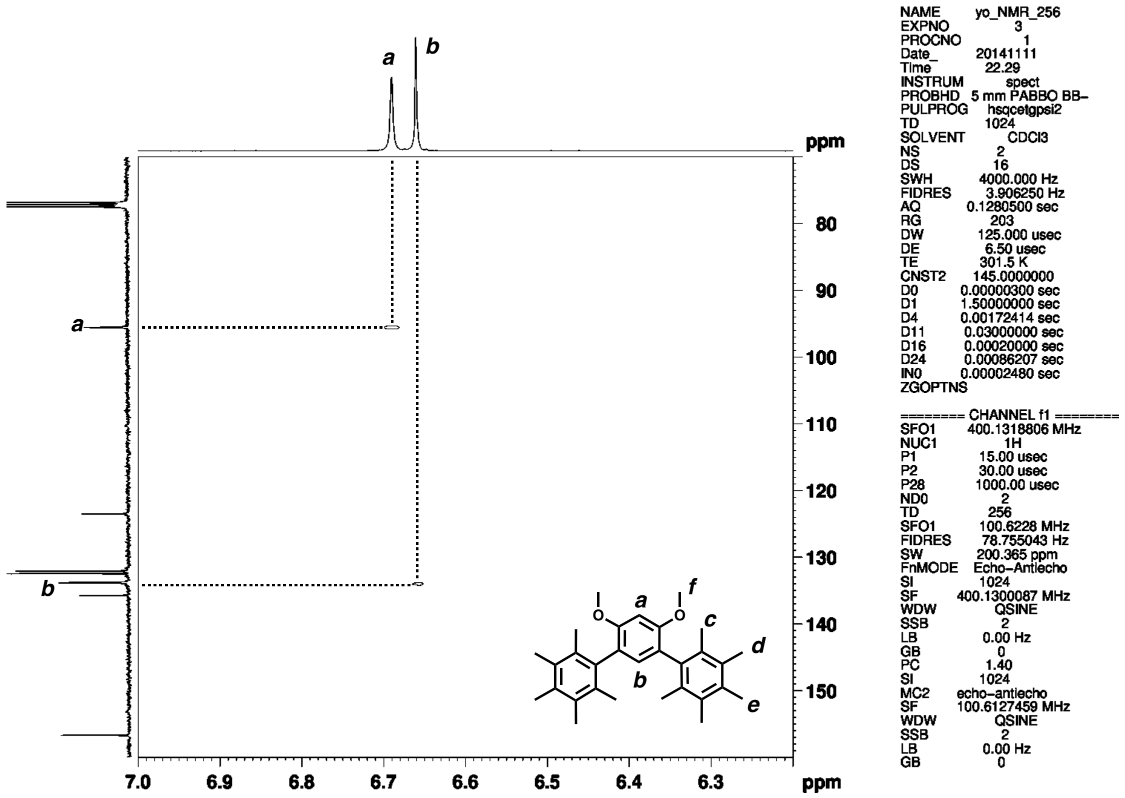

Figure S4a. HSQC spectrum (400 MHz, $CDCl_3$, r.t.) of **4a**.

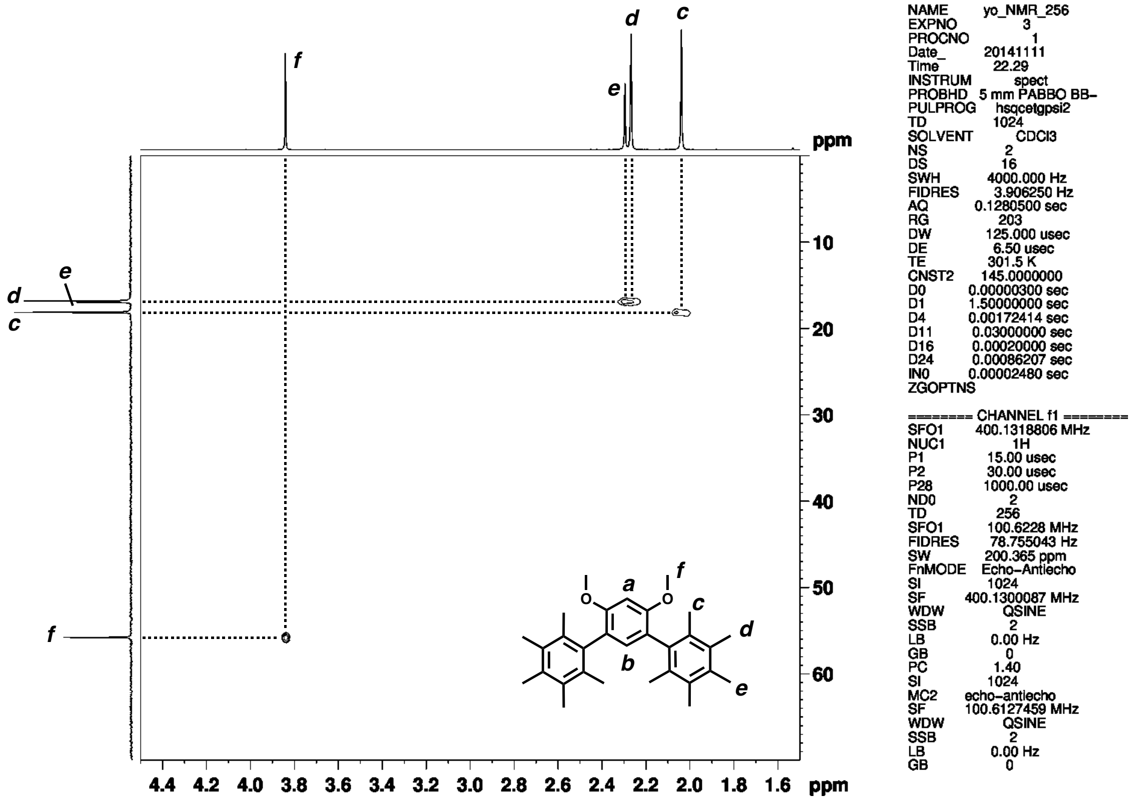

Figure S4b. HSQC spectrum (400 MHz, $CDCl_3$, r.t.) of **4a**.

Synthesis of 5a YO-101

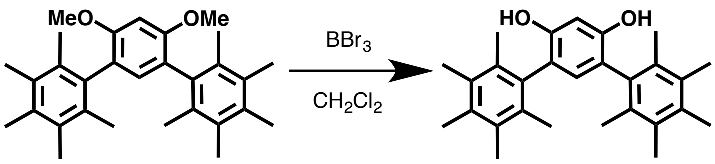

Pentamethylbenzene dimer **4a** (0.500 g, 1.16 mmol) and dry CH_2Cl_2 (50 mL) were added to a 200 mL glass flask. A CH_2Cl_2 solution (1.0 M) of BBr_3 (4.6 mL, 4.6 mmol) was added dropwise to this flask at 0 ºC under N_2. The reaction mixture was stirred and allowed to warm to r.t. overnight. The reaction was quenched with H_2O (50 mL). The two layers were separated and the aqueous layer was extracted with CH_2Cl_2 (3 × 100 mL). The combined organic layers were dried over $MgSO_4$, filtrated, and concentrated under reduced pressure. The crude product was washed with acetone and hexane to afford **5a** (0.453 g, 1.13 mmol, 97% yield) as a white solid.[2]

^{1}H NMR (400 MHz, $CDCl_3$, r.t.): δ 2.05 (s, 12H), 2.26 (s, 12H), 2.29 (s, 6H), 4.62 (s, 6H), 6.60 (s, 1H), 6.67 (s, 1H). ^{13}C NMR (100 MHz, $CDCl_3$, r.t.): δ 16.9 (CH_3), 16.9 (CH_3), 17.9 (CH_3), 101.6 (CH), 121.1 (C_q), 131.5 (C_q), 132.1 (C_q), 133.2 (C_q), 133.8 (CH), 135.3 (C_q), 153.0 (C_q). FT-IR (KBr, cm^{-1}): 3518, 2986, 2925, 2872, 1630, 1595, 1494, 1453, 1418, 1376, 1336, 1258, 1213, 1131, 1099, 1066, 1019, 902, 851, 783, 604. MALDI-TOF MS (dithranol): *m/z* Calcd. for $C_{28}H_{34}O_2$: 402.26, Found 402.16 $[M]^+$. E.A.: Calcd. for $C_{28}H_{34}O_2$•0.6CH_3COCH_3: C, 81.83; H, 8.66. Found: C, 81.67; H, 8.52.

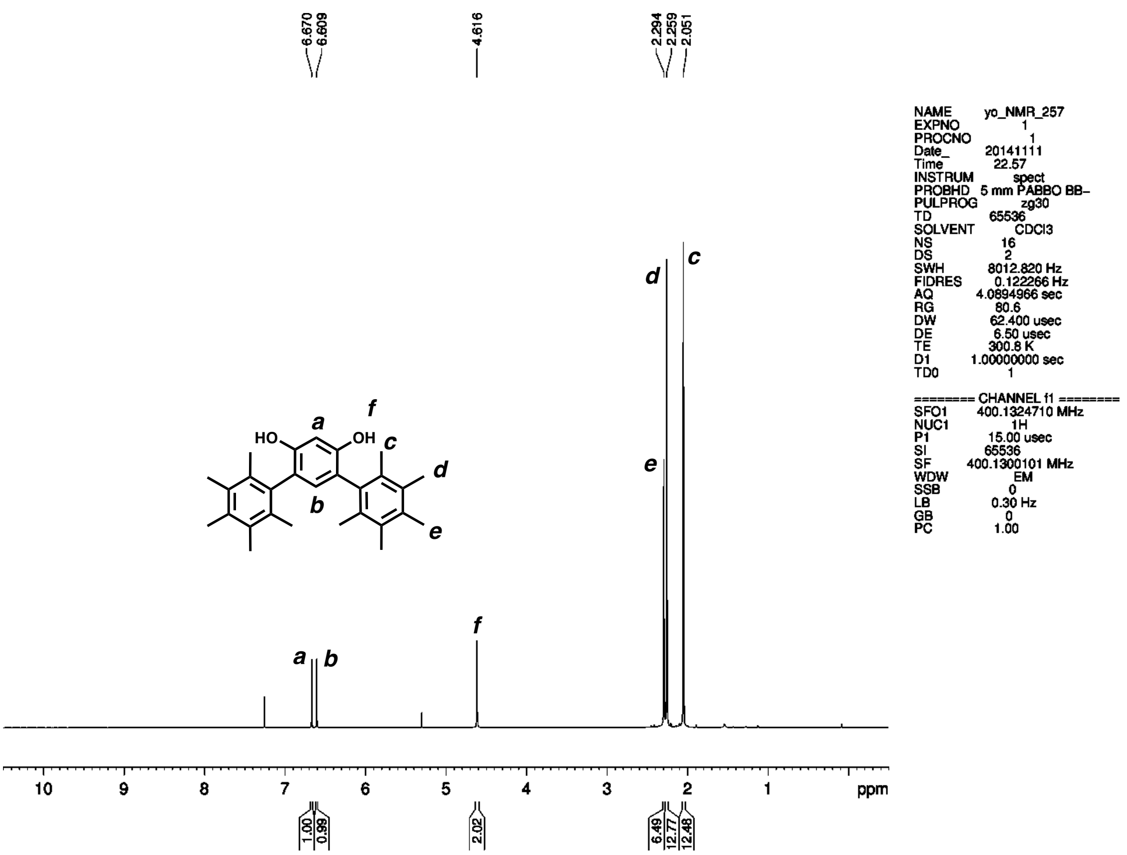

Figure S5. ^{1}H NMR spectrum (400 MHz, $CDCl_3$, r.t.) of **5a**.

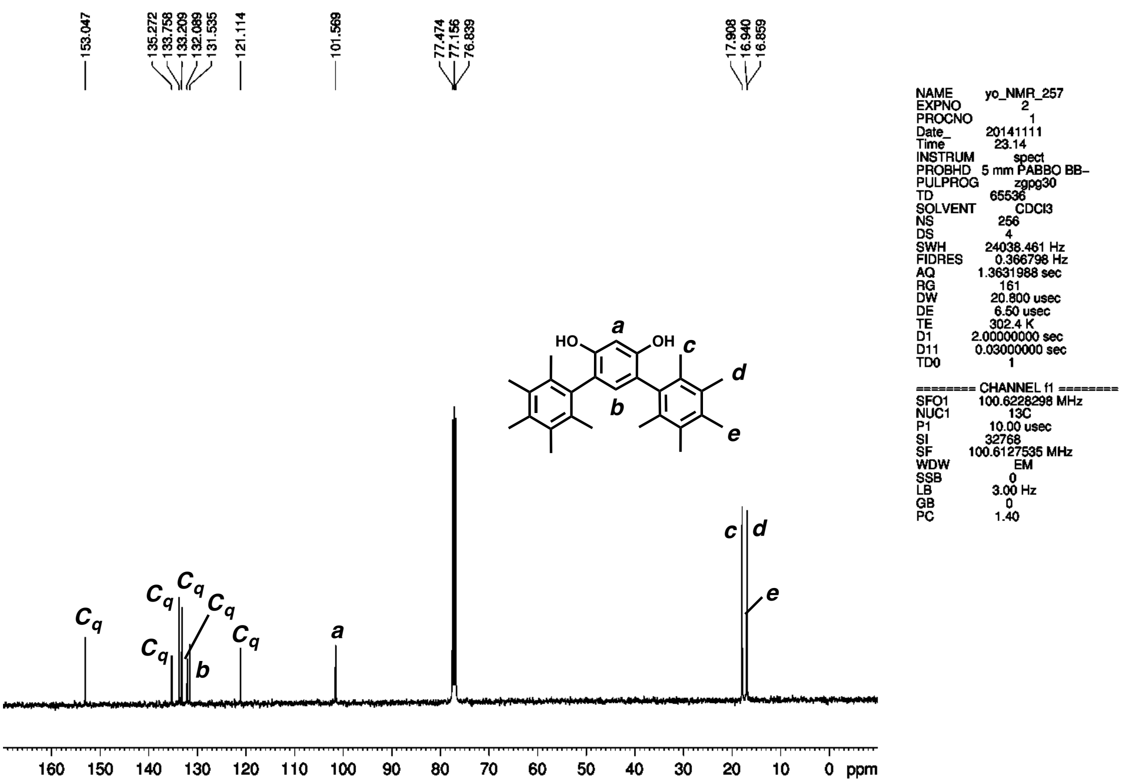

Figure S6. ^{13}C NMR spectrum (100 MHz, $CDCl_3$, r.t.) of **5a**.

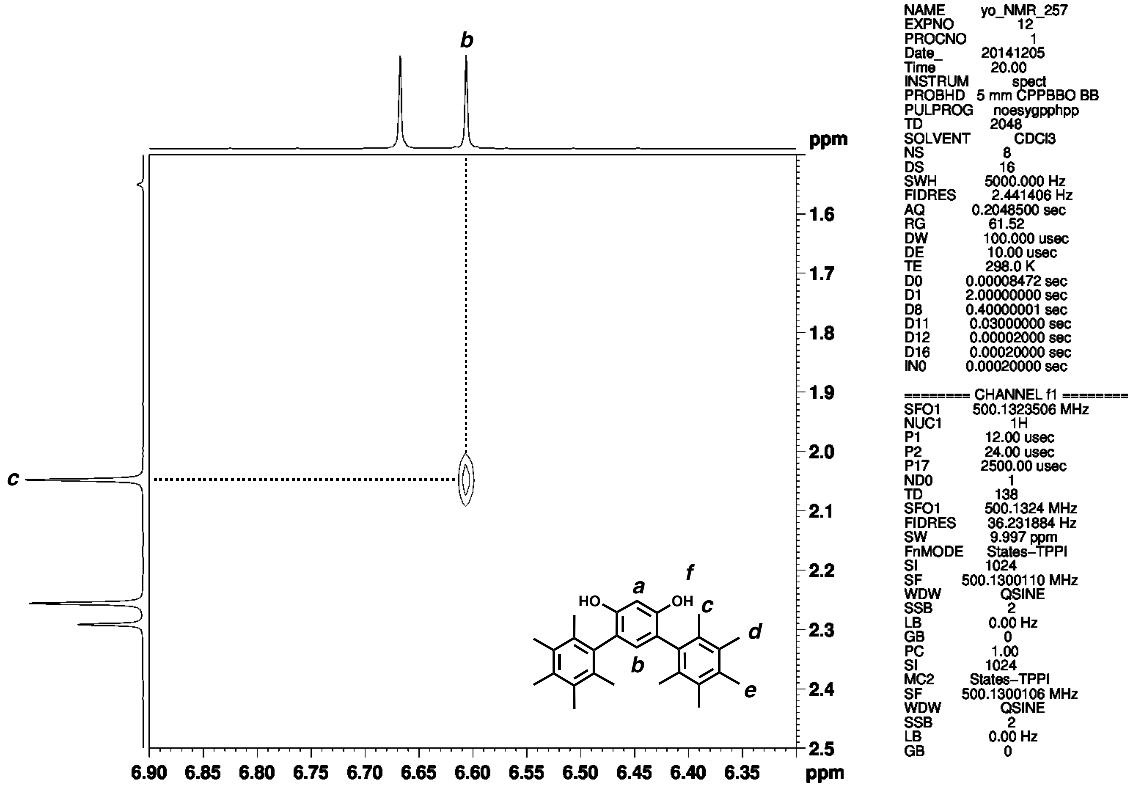

Figure S7a. NOESY spectrum (400 MHz, $CDCl_3$, r.t.) of **5a**.

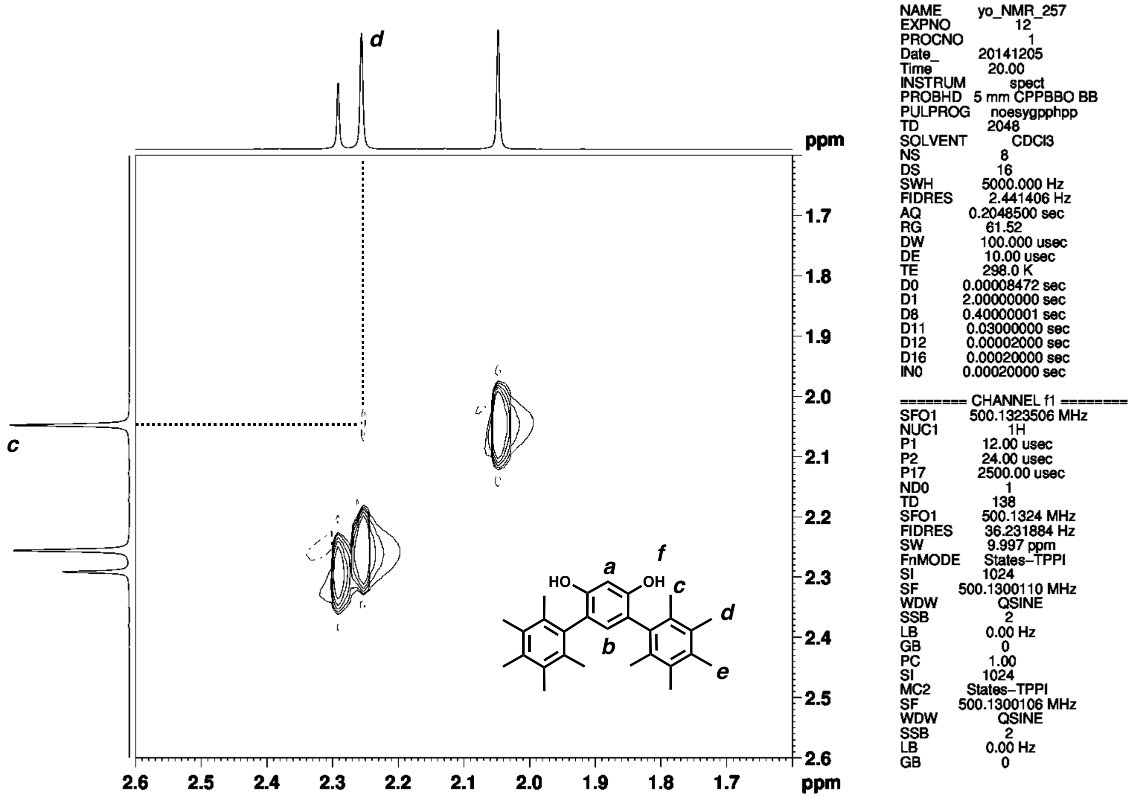

Figure S7b. NOESY spectrum (400 MHz, $CDCl_3$, r.t.) of **5a**.

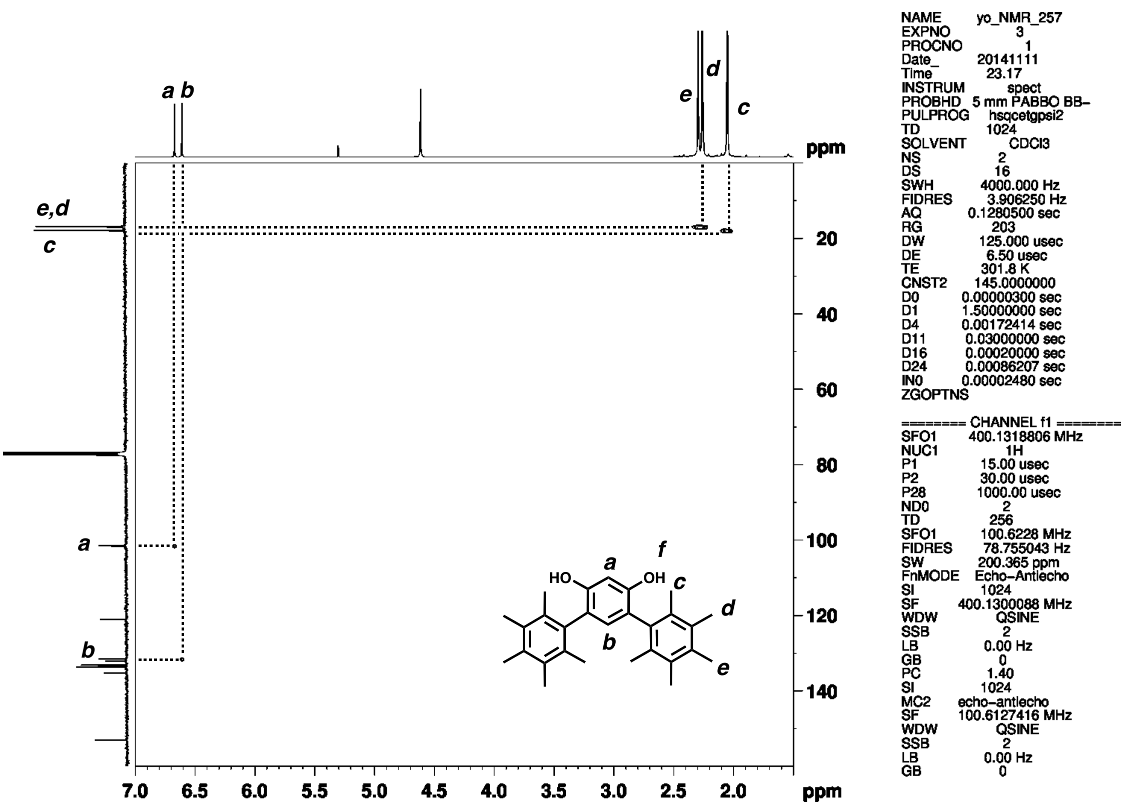

Figure S8. HSQC spectrum (400 MHz, $CDCl_3$, r.t.) of **5a.**

Synthesis of 1a YO-164

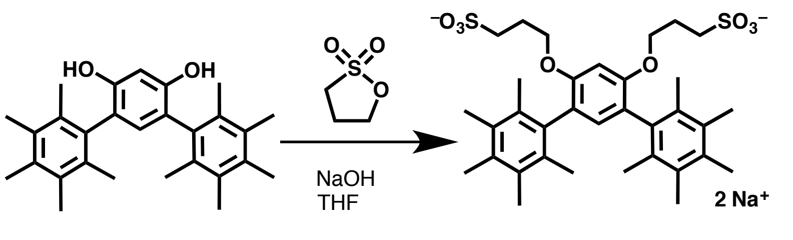

Pentamethylbenzene dimer **5a** (100 mg, 0.248 mmol), NaOH (60 mg, 1.5 mmol), and THF (30 mL) were added to a 50 mL glass flask. 1,3-Propanesultone (91 mg, 0.74 mmol) was added dropwise to this flask. The resultant mixture was stirred at r.t. overnight. The solvent was filtrated and then CH_2Cl_2 and hexane was added to the filtrate. The resultant solution was concentrated under reduced pressure. The crude product was washed with water and 1-propanol to afford **1a** (0.108 g, 0.156 mmol, 63% yield) as a white solid.

^{1}H NMR (400 MHz, CD_3OD, r.t.): δ 1.96 (s, 12H), 2.06 (dt, J = 6.8, 6.0 Hz, 4H), 2.22 (s, 12H), 2.25 (s, 6H), 2.70 (t, J = 6.8 Hz, 4H), 4.06 (t, J = 6.0 Hz, 4H), 6.42 (s, 1H), 6.81

(s, 1H). ^{13}C NMR (125 MHz, $CDCl_3$, r.t.): δ 16.8 (CH_3), 18.4 (CH_3), 26.4 (CH_2), 49.0 (CH_2), 68.6 (CH_2), 100.3 (CH), 126.0 (C_q), 132.7 (C_q), 133.0 (C_q), 134.1 (CH), 134.2 (C_q), 137.0 (C_q), 157.1 (C_q). FT-IR (KBr, cm^{-1}): 3446, 2925, 1630, 1604, 1577, 1502, 1452, 1385, 1323, 1274, 1186, 1108, 1049, 797, 739, 610. HR MS (ESI): Calcd. for $C_{34}H_{44}O_8S_2$ 322.1233, Found 322.1223 $[M–2Na^+]^{2-}$. E.A.: Calcd. for $C_{34}H_{44}Na_2O_8S_2$•2.5CH_2Cl_2: C, 48.54; H, 5.47. Found: C, 48.40; H, 5.55.

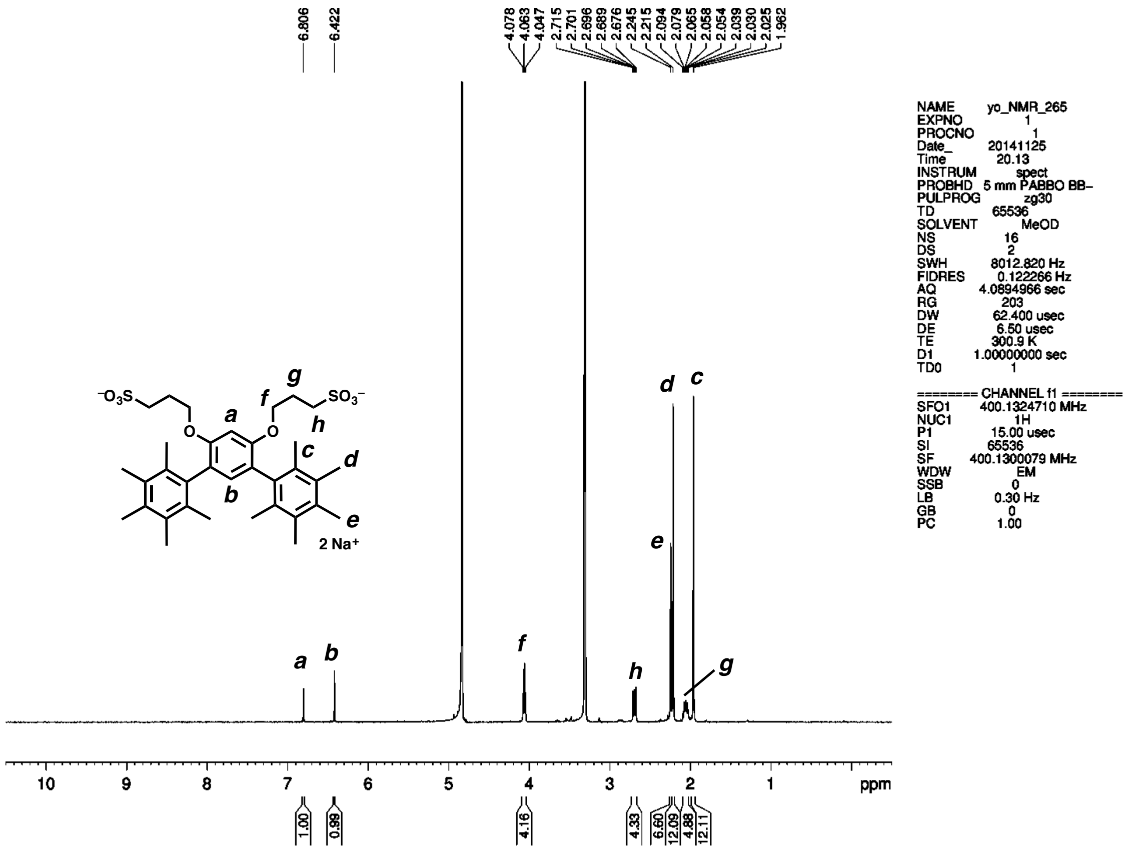

Figure S9. ^1H NMR spectrum (400 MHz, CD_3OD, r.t.) of **1a**.

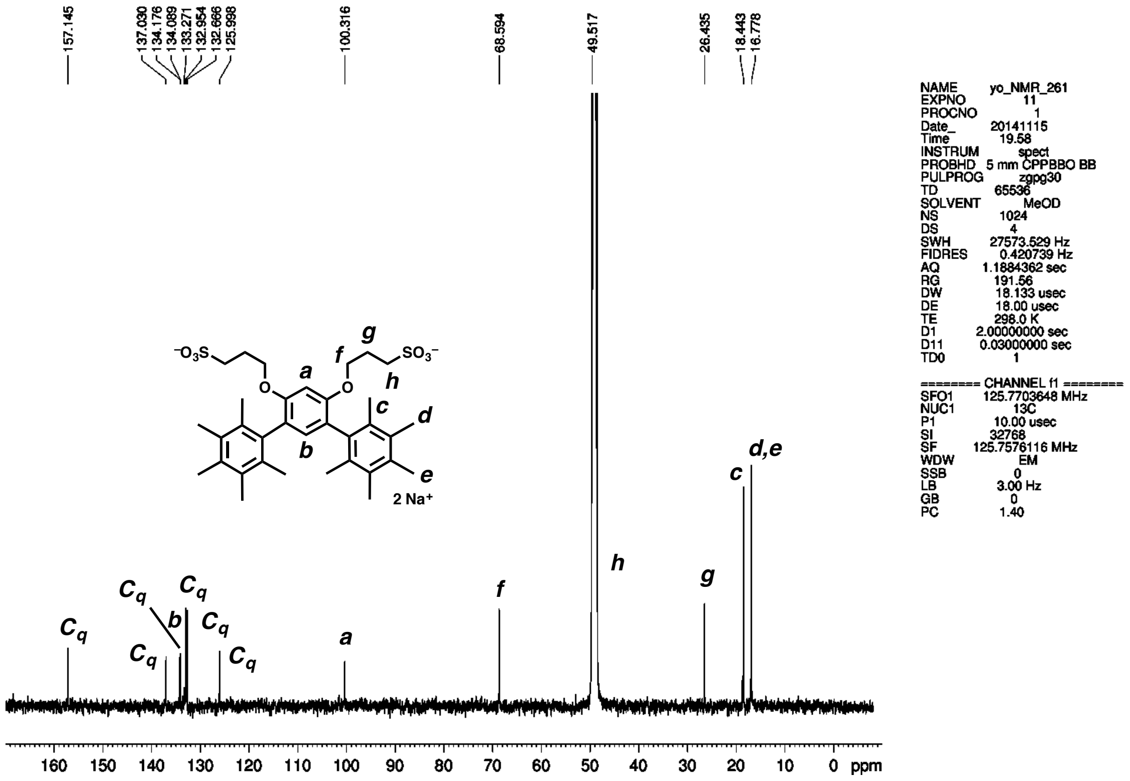

Figure S10. ^{13}C NMR spectrum (125 MHz, CD_3OD, r.t.) of **1a**.

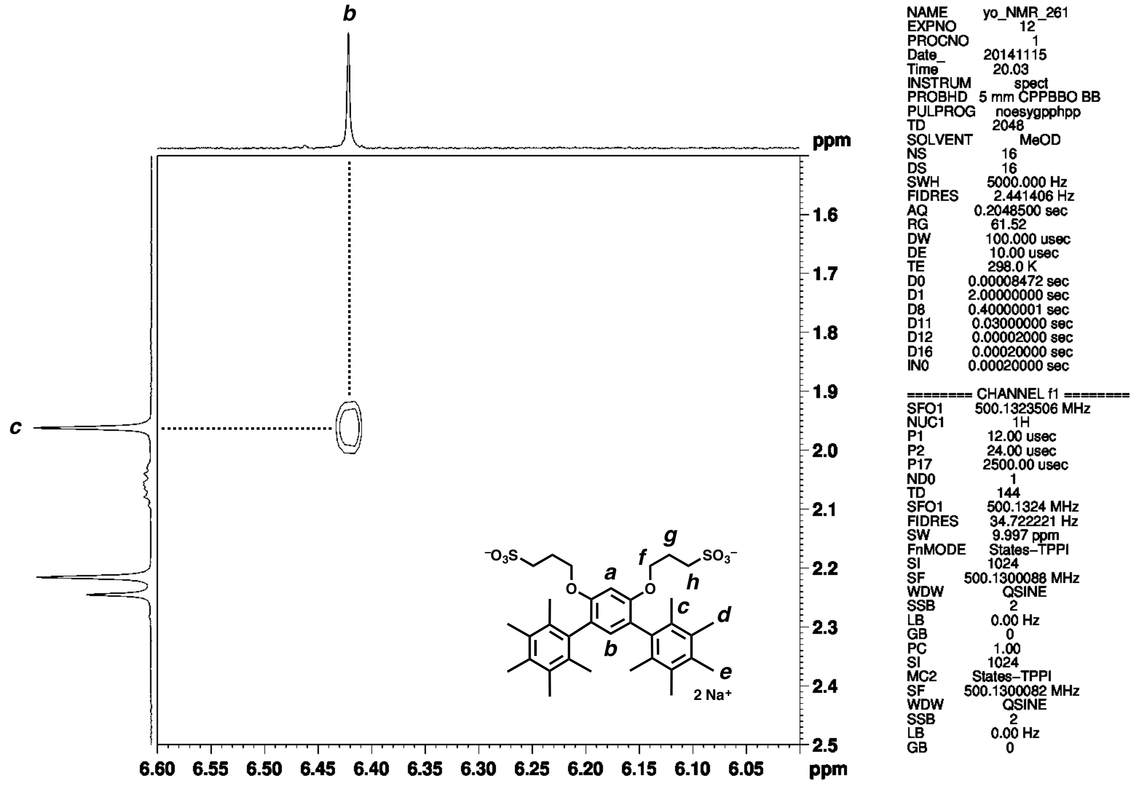

Figure S11a. NOESY spectrum (500 MHz, CD_3OD, r.t.) of **1a**.

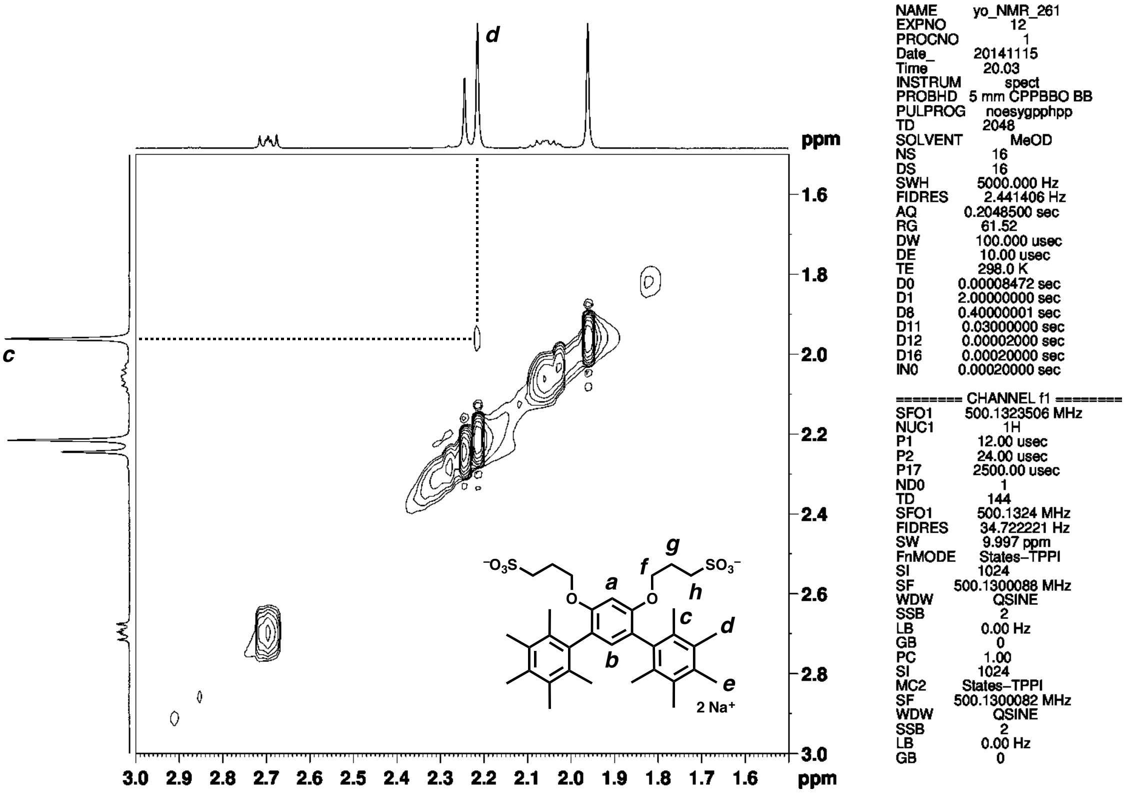

Figure S11b. NOESY spectrum (500 MHz, CD_3OD, r.t.) of **1a**.

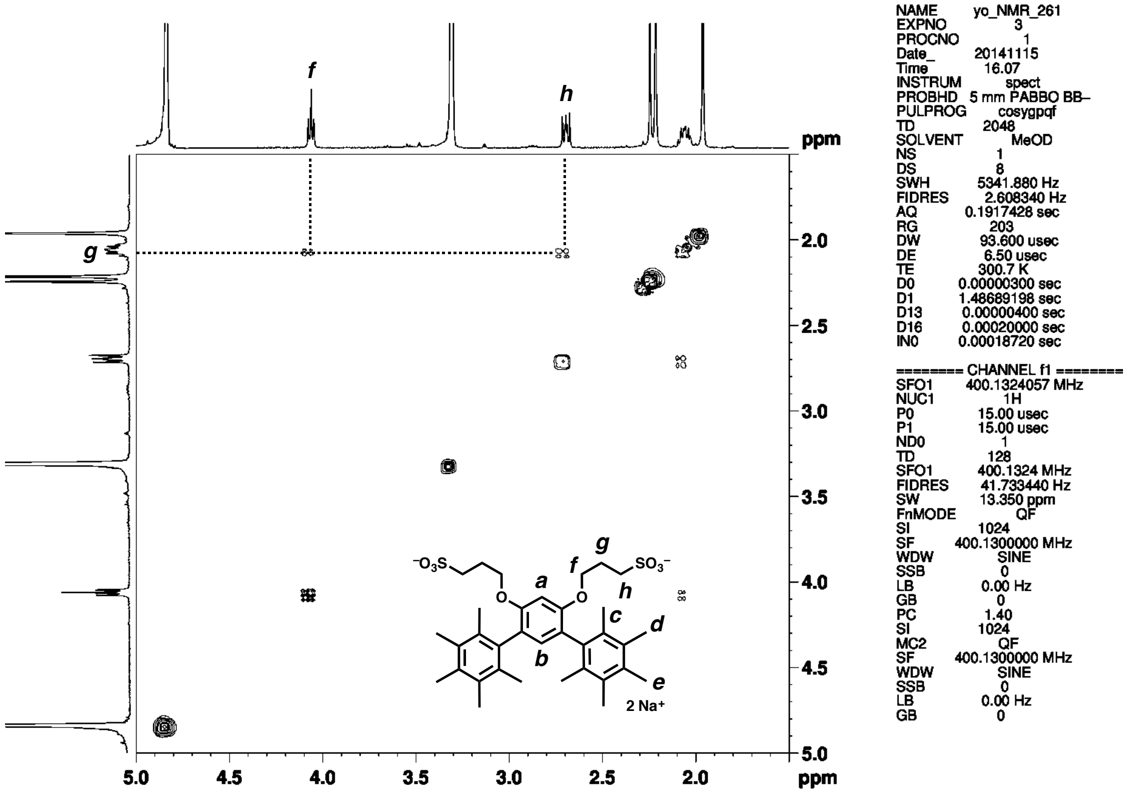

Figure S12. ^{1}H-^{1}H COSY spectrum (400 MHz, CD_3OD, r.t.) of **1a**.

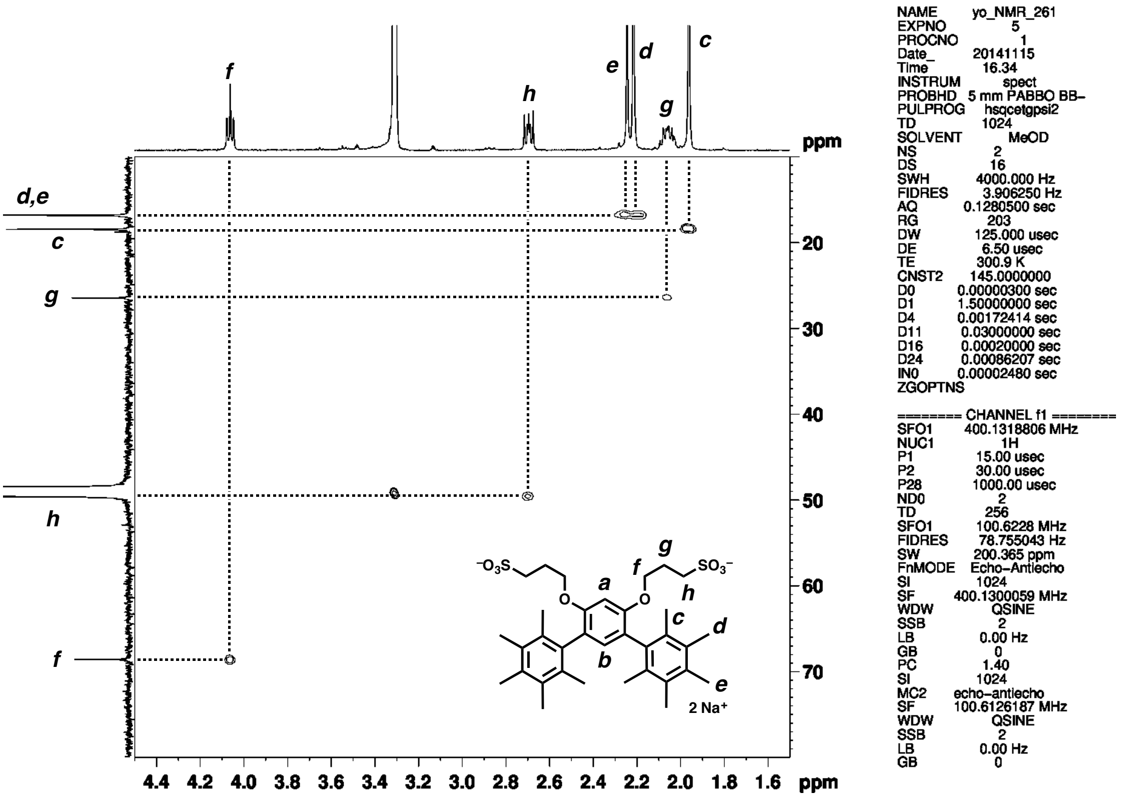

Figure S13a. HSQC spectrum (400 MHz, CD_3OD, r.t.) of **1a.**

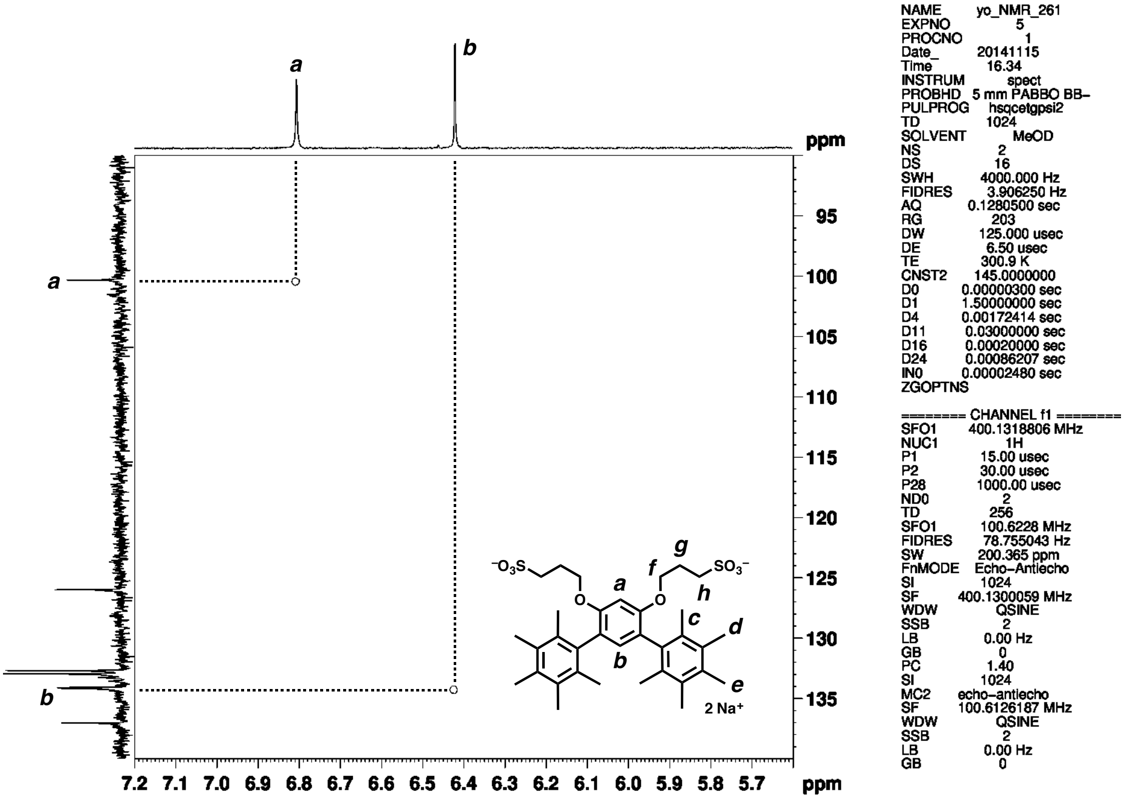

Figure S13b. HSQC spectrum (400 MHz, CD_3OD, r.t.) of **1a.**

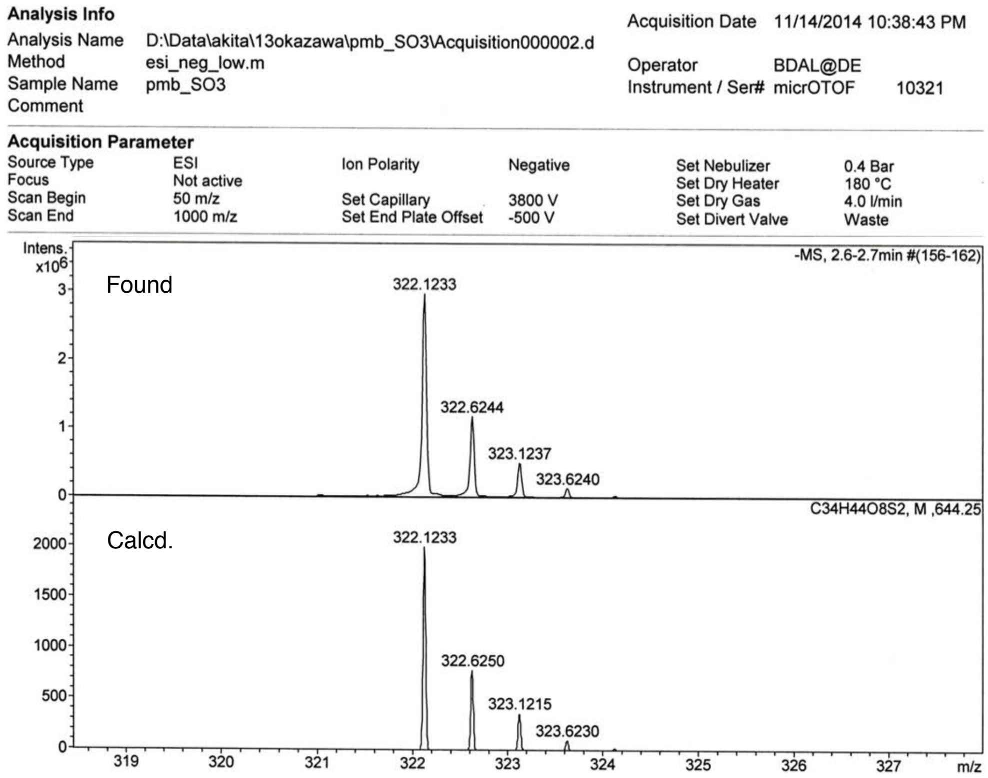

Figure S14. HR MS spectrum (ESI) of **1a**.

Self-assembly of 2a YO-104

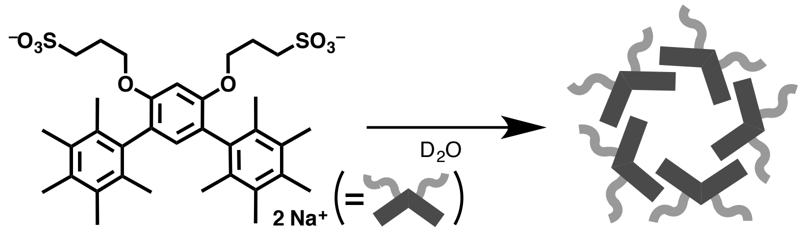

Amphiphile **1a** (5.5 mg, 8.0 μmol) and D_2O (0.4 mL) were added to a glass test tube. When the mixture was stirred at r.t. for 1 min, the formation of **2a** was confirmed by NMR, DLS, and AFM analyses.

^{1}H NMR (500 MHz, D_2O, 2.0 mM based on **1a**, r.t.): δ 1.97 (s, 12H), 2.00 (dt, J = 6.4, 6.0 Hz, 4H), 2.22 (s, 12H), 2.27 (s, 6H), 2.62 (m, J = 6.4 Hz, 4H), 4.09 (t, J = 6.0 Hz, 4H), 6.58 (s, 1H), 6.96 (s, 1H). ^{13}C NMR (125 MHz, D_2O, 2.0 mM based on **1a**, r.t.): δ 15.7 (CH_3), 15.9 (CH_3), 17.4 (CH_3), 24.3 (CH_2), 47.6 (CH_2), 68.2 (CH_2), 101.9 (CH), 125.9 (C_q), 132.4 (C_q), 132.8 (C_q), 133.0 (CH), 134.5 (C_q), 135.0 (C_q), 155.0 (C_q). DOSY NMR (400 MHz, D_2O, 2.0 based on **1a**, 25 ºC): D = 4.16 x 10^{-10} m^2 s^{-1}.

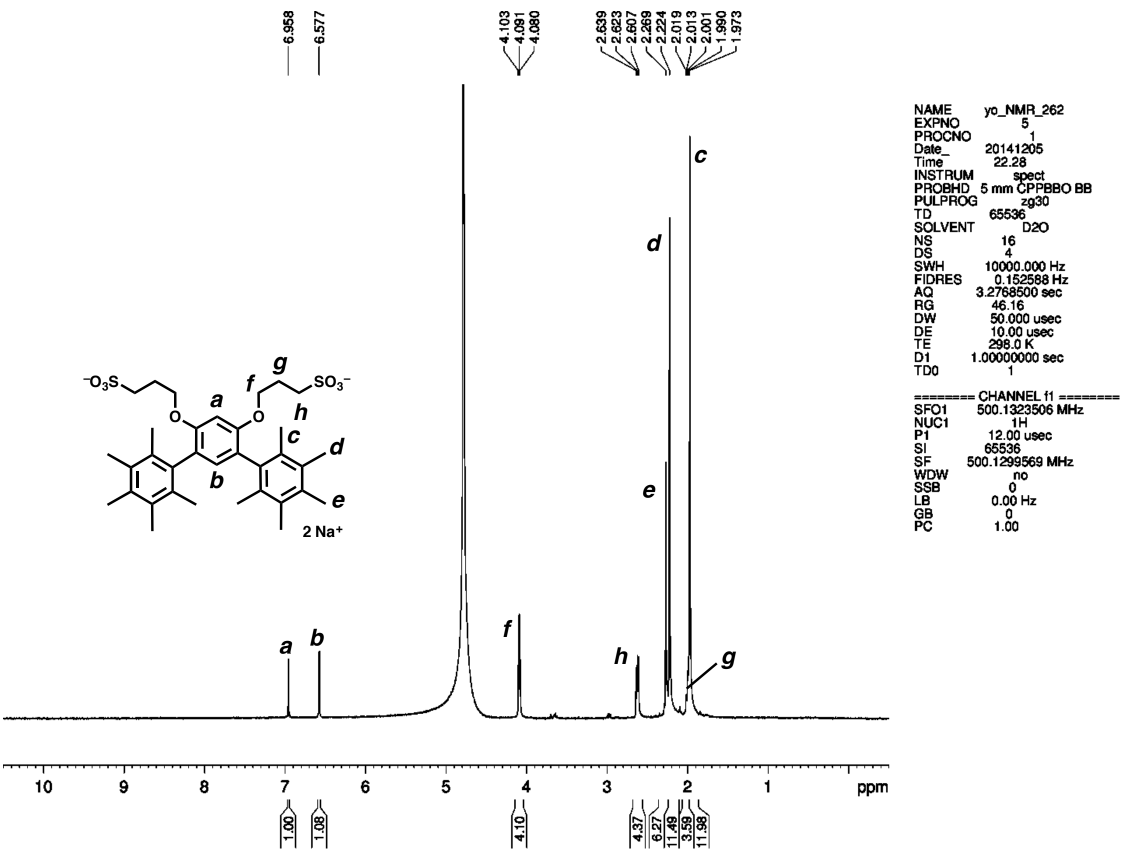

Figure S15. ^{1}H NMR spectrum (500 MHz, D_2O, 2.0 mM based on **1a**, r.t.) of **2a**.

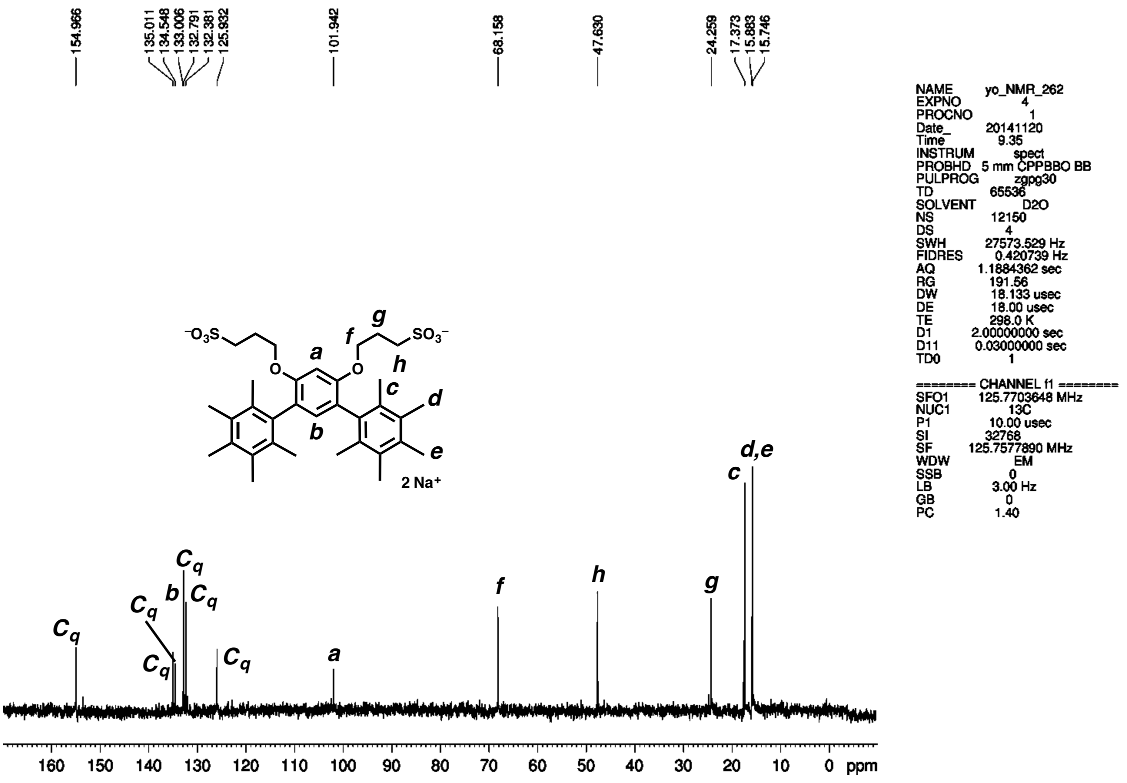

Figure S16. ^{13}C NMR spectrum (125 MHz, D_2O, 2.0 mM based on **1a**, r.t.) of **2a**.

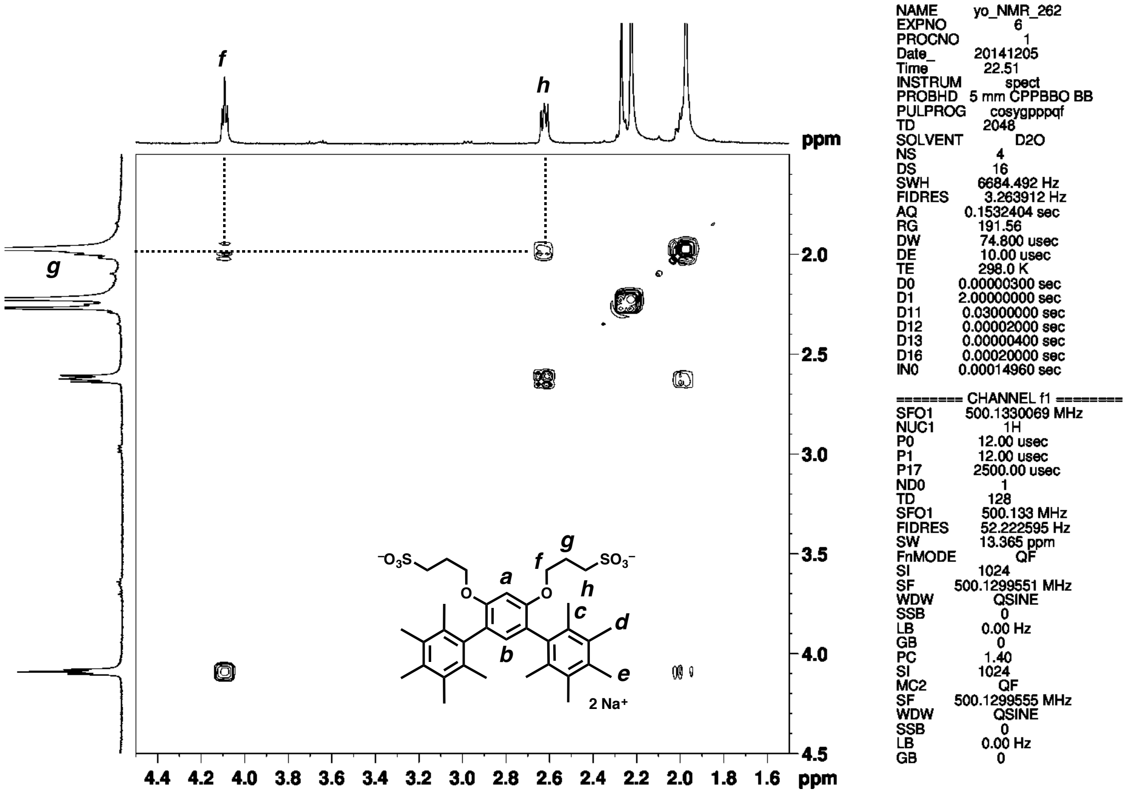

Figure S17. ^{1}H-^{1}H COSY spectrum (400 MHz, D_2O, 2.0 mM based on **1a**, r.t.) of **2a**.

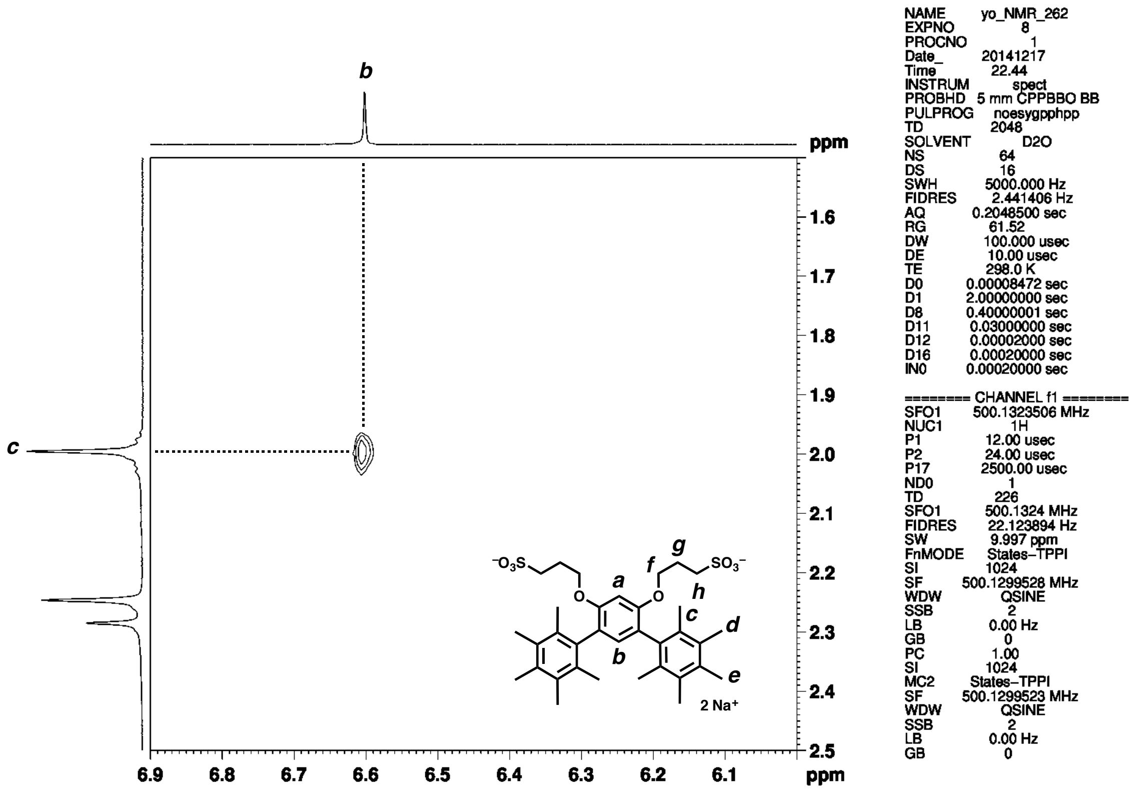

Figure S18a. NOESY spectrum (500 MHz, D_2O, 2.0 mM based on **1a**, r.t.) of **2a**.

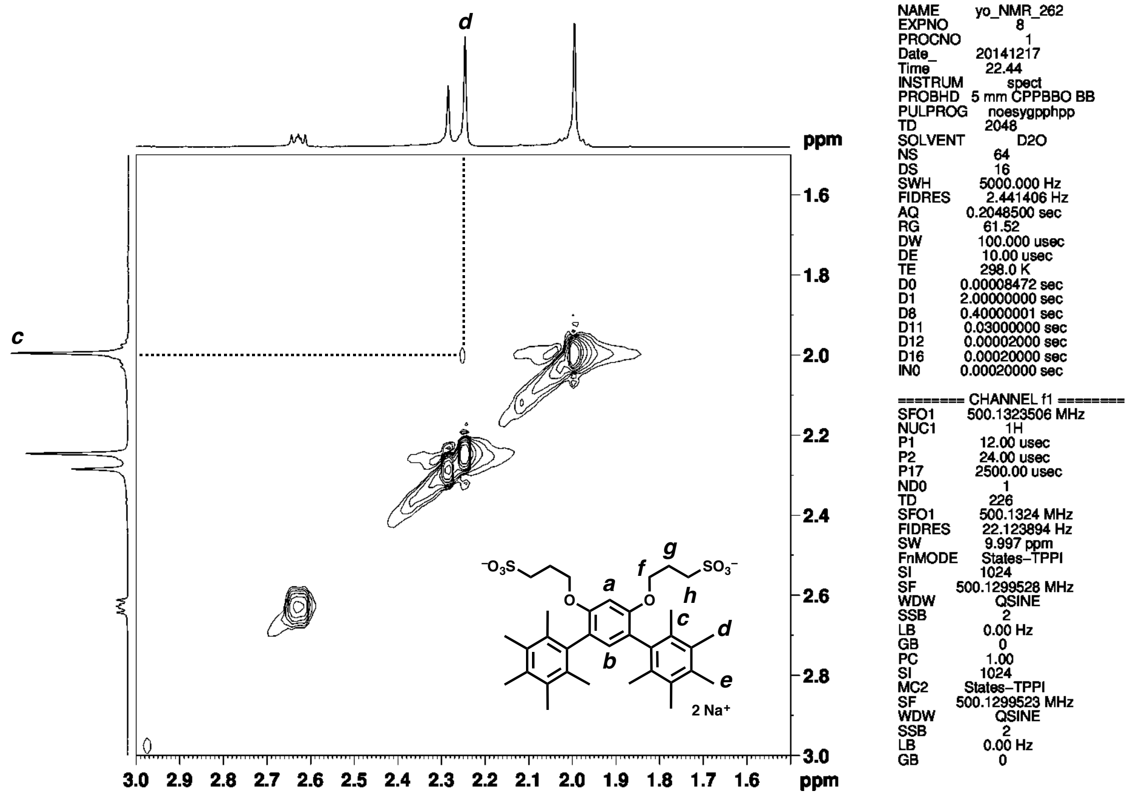

Figure S18b. NOESY spectrum (500 MHz, D_2O, 2.0 mM based on **1a**, r.t.) of **2a**.

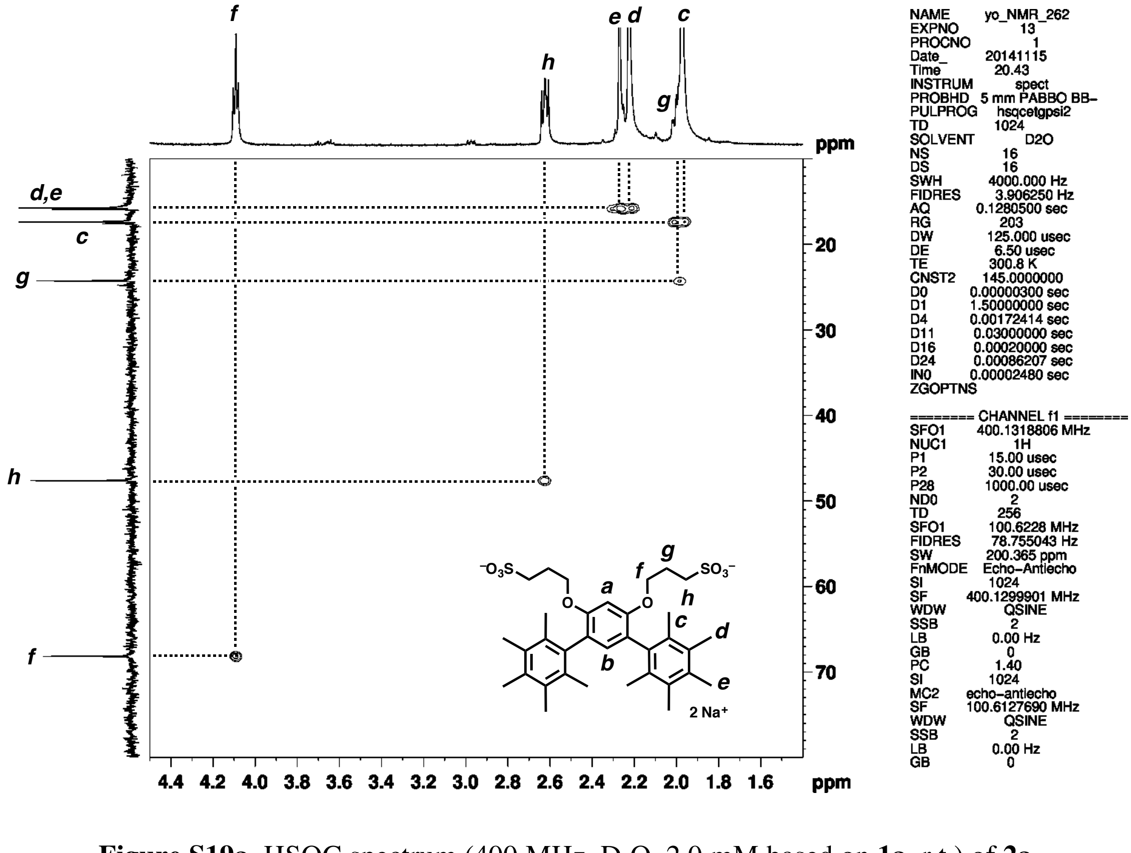

Figure S19a. HSQC spectrum (400 MHz, D_2O, 2.0 mM based on **1a**, r.t.) of **2a**.

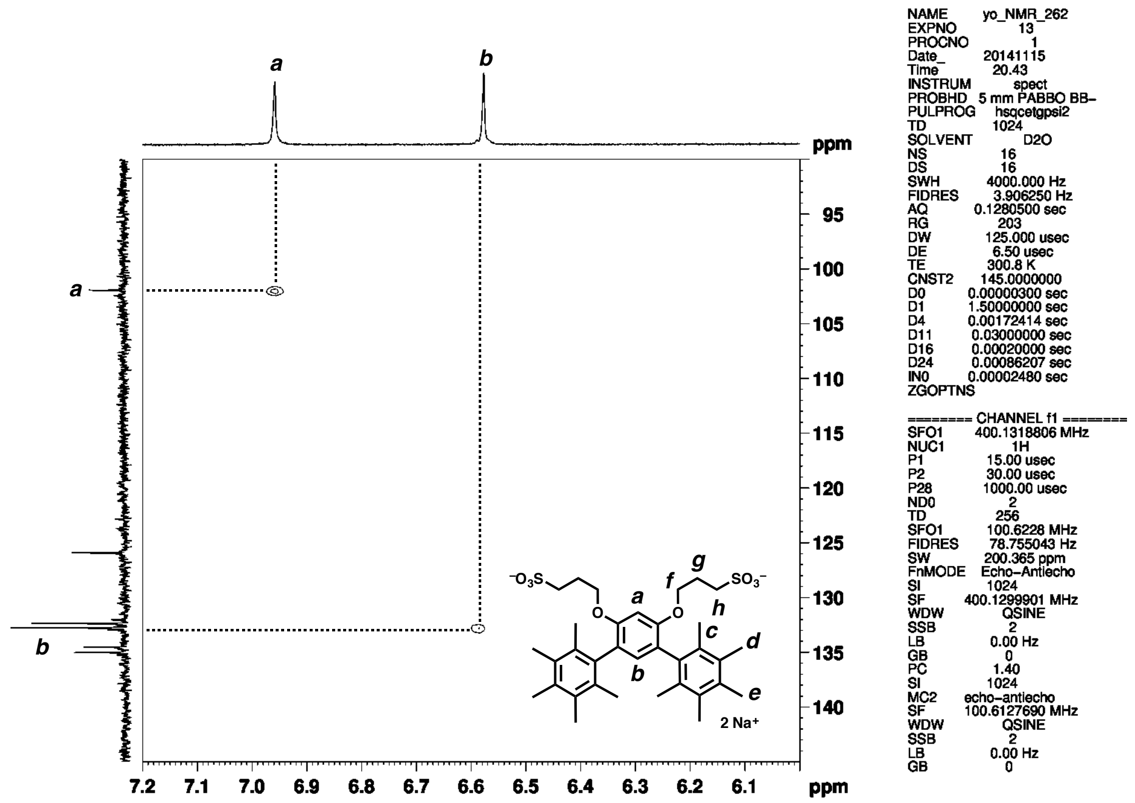

Figure S19b. HSQC spectrum (400 MHz, D_2O, 2.0 mM based on **1a**, r.t.) of **2a**.

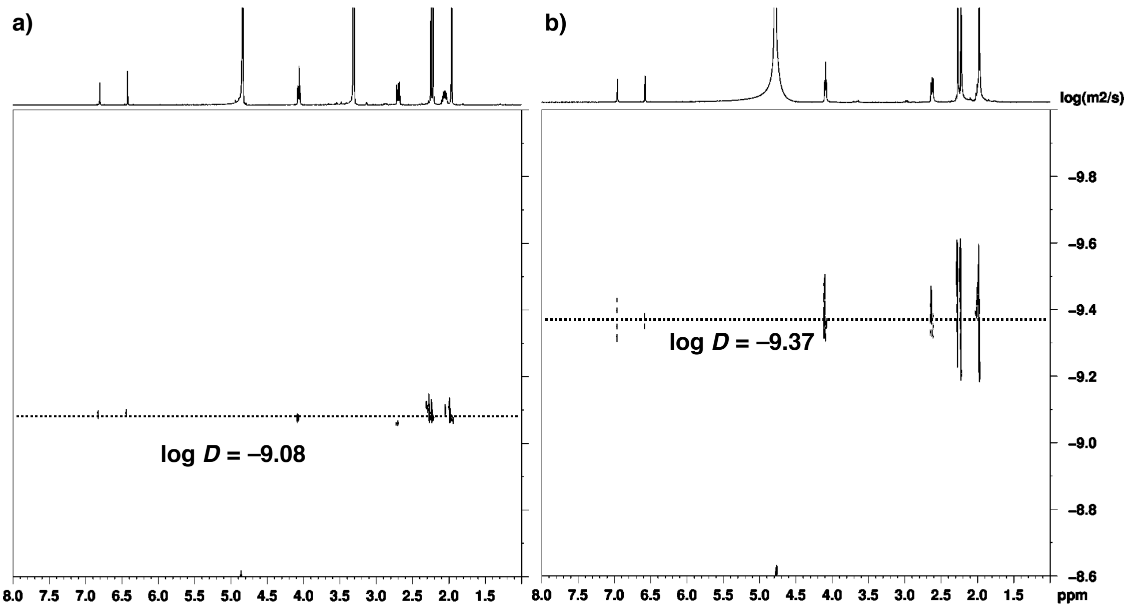

Figure S20. DOSY NMR spectra (400 MHz, 25 °C) of a) **1a** in CD_3OD and b) **2a** in D_2O (2.0 mM based on **1a**).

Synthesis of 4b YO-162

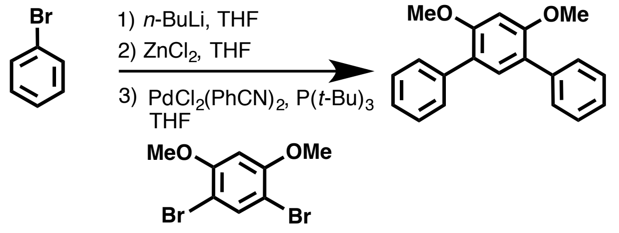

1-Bromobenzene (2.000 g, 12.73 mmol) and dry THF (50 mL) were added to a 2-necked 300 mL glass flask filled with N_2. A hexane solution (2.69 M) of *n*-butyllithium (5.2 mL, 14 mmol) was added dropwise to the flask at –80 ºC under N_2. After stirring at –80 ºC for 2 h, a dry THF solution (30 mL) of $ZnCl_2$ (2.250 g, 16.55 mmol) was added to the solution. The resultant mixture was further stirred at –80 ºC for 1 h and then the solution was warmed to r.t. for 1 d to obtain phenylzinc chloride. 1,5-Dibromo-2,4-dimethoxybenene (1.538 g, 5.197 mmol), $PdCl_2(PhCN)_2$ (140 mg, 0.364 mmol), and dry THF (30 mL) were added to a 50 mL glass flask filled with N_2. A hexane solution (0.95 M) of tri-*tert*-butylphosphine (0.7 mL, 0.7 mmol) was added to the 50 mL flask. After stirring for 30 min at r.t., the mixture was added to the 300 mL flask and then the resulted solution was further stirred at 85 ºC for 2 d. The precipitated crude product was extracted with CH_2Cl_2 and hexane (as an azeotropic solvent). The solution was concentrated under reduced pressure. The crude product was collected and washed with CH_3OH to afford **4b** (0.962 g, 3.31 mmol, 64% yield) as a white solid.

^{1}H NMR (400 MHz, $CDCl_3$, r.t.): δ 3.92 (s, 6H), 6.72 (s, 1H), 7.35 (t, J = 7.6 Hz, 2H), 7.45 (dd, J = 8.0, 7.6 Hz, 4H), 7.61 (d, J = 8.0 Hz, 4H). ^{13}C NMR (100 MHz, $CDCl_3$, r.t.): δ 56.0 (CH_3), 96.6 (CH), 123.5 (C_q), 126.7 (CH), 128.1 (CH), 129.6 (CH), 133.1 (CH), 138.3 (C_q), 157.0 (C_q). FT-IR (KBr, cm^{-1}): 3444, 3021, 3001, 2928, 2836, 1955, 1611, 1582, 122, 1483, 1467, 1433, 1388, 1311, 1284, 1261, 1240, 1203, 1181, 1165, 1075, 1052, 1031, 1011, 948, 899, 818, 765, 723, 701, 678. GC-MS (*m/z*): Calcd. for $C_{20}H_{18}O_2$: 290, Found 290 $[M]^+$. E.A.: Calcd. for $C_{20}H_{18}O_2$•0.4CH_3OH: C, 80.82; H, 6.52. Found: C, 80.99; H, 6.45.

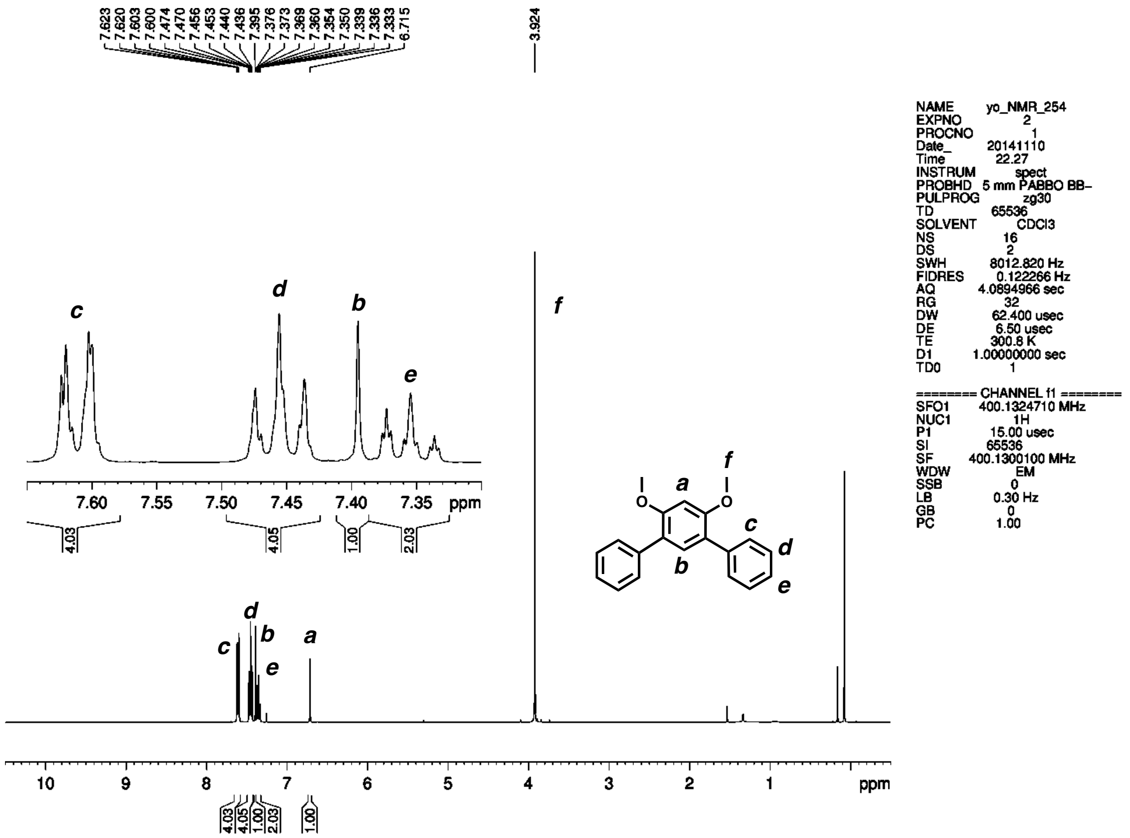

Figure S21. ^{1}H NMR spectrum (400 MHz, $CDCl_3$, r.t.) of **4b**.

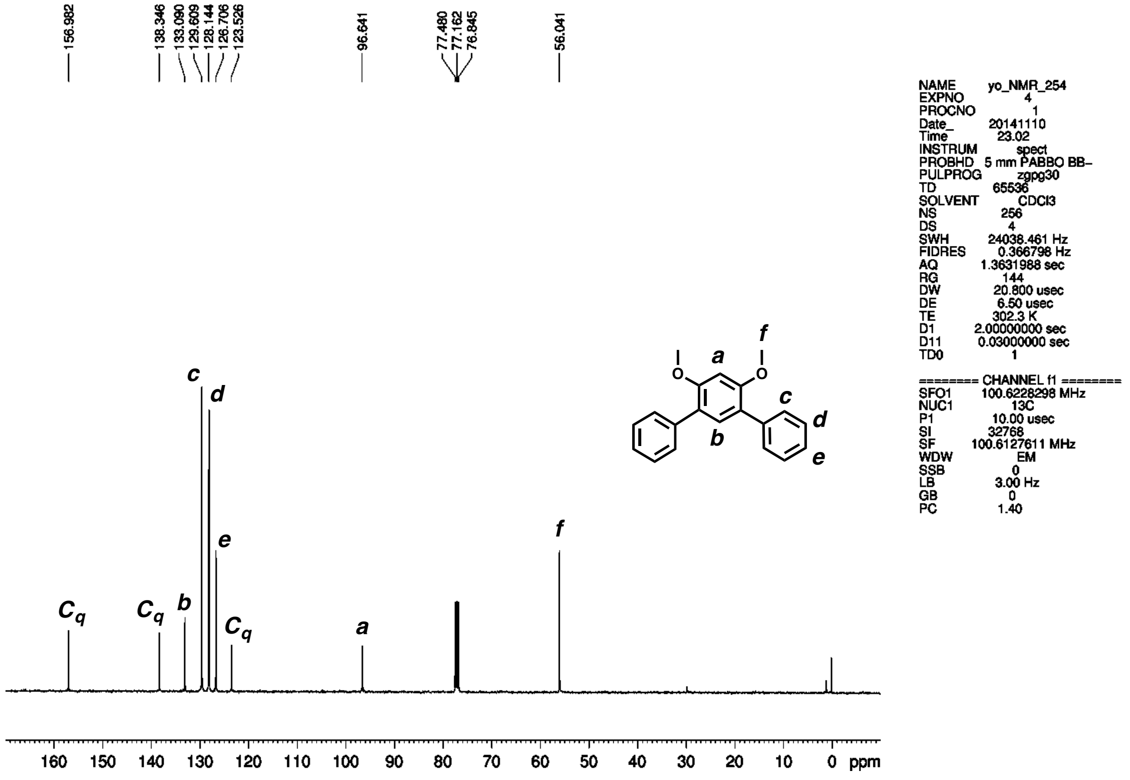

Figure S22. ^{13}C NMR spectrum (100 MHz, $CDCl_3$, r.t.) of **4b**.

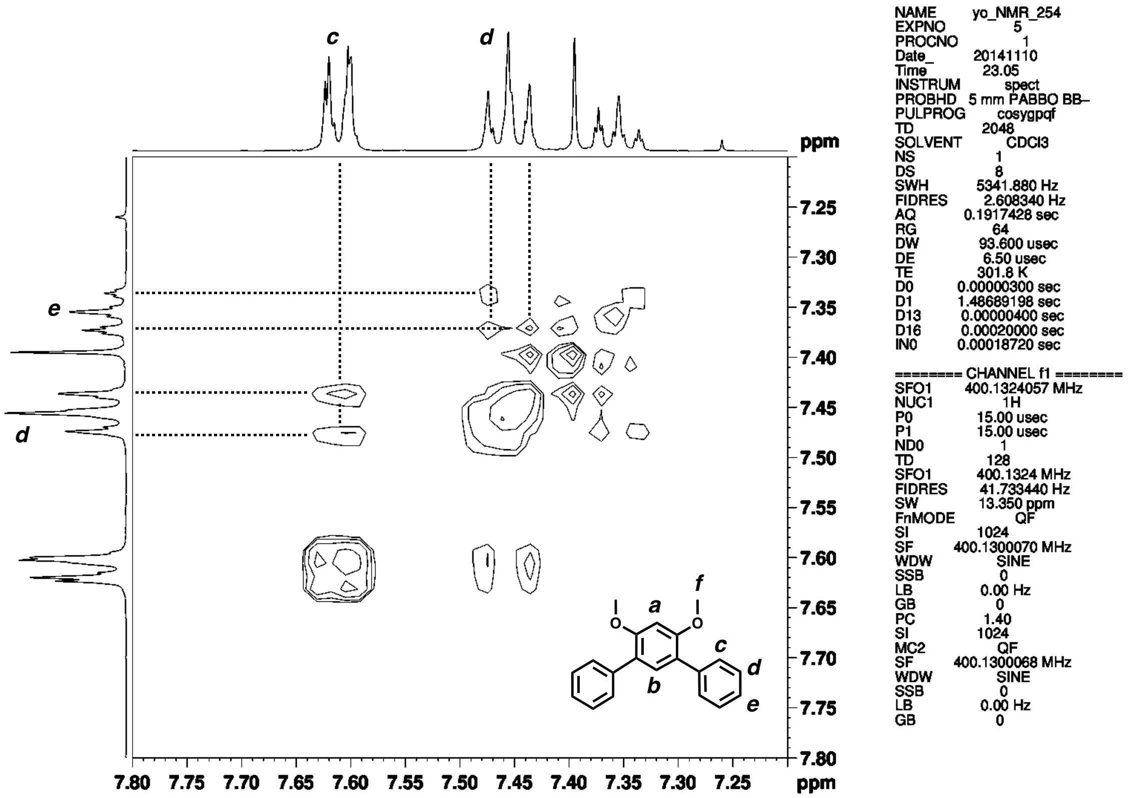

Figure S23. ^{1}H-^{1}H COSY spectrum (400 MHz, $CDCl_3$, r.t.) of **4b**.

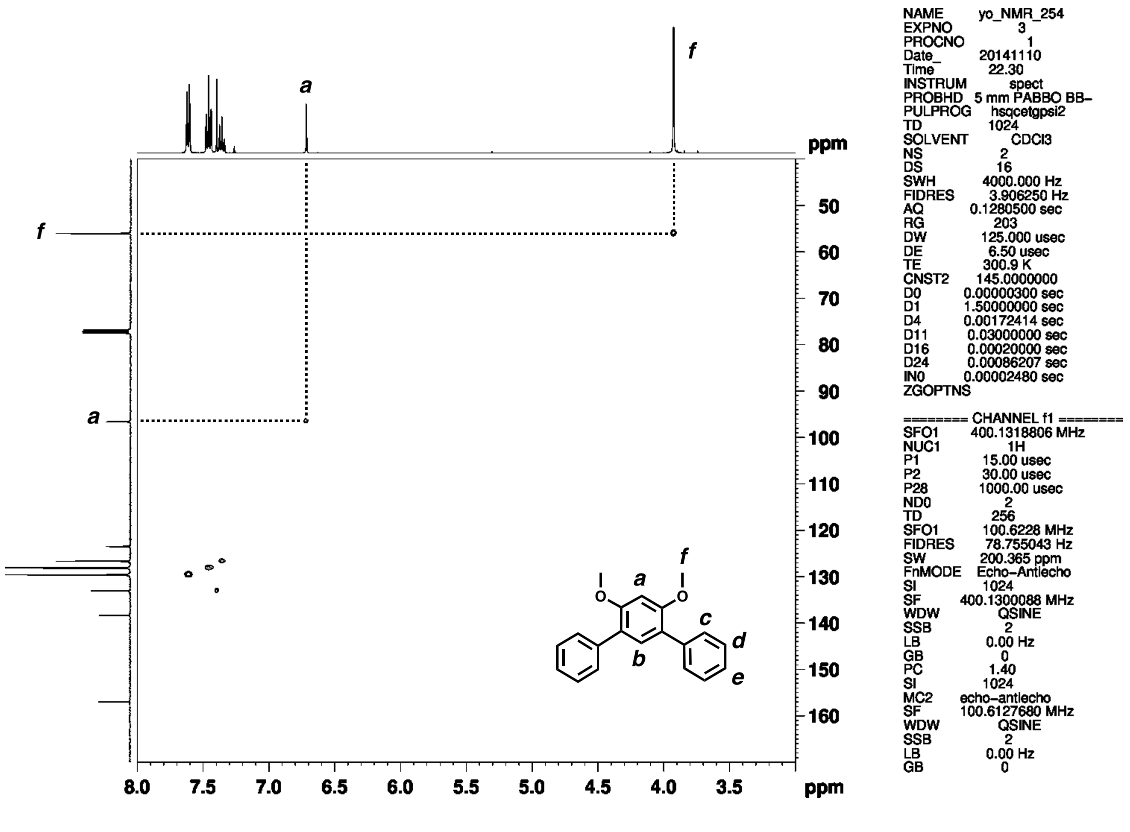

Figure S24a. HSQC spectrum (400 MHz, $CDCl_3$, r.t.) of **4b**.

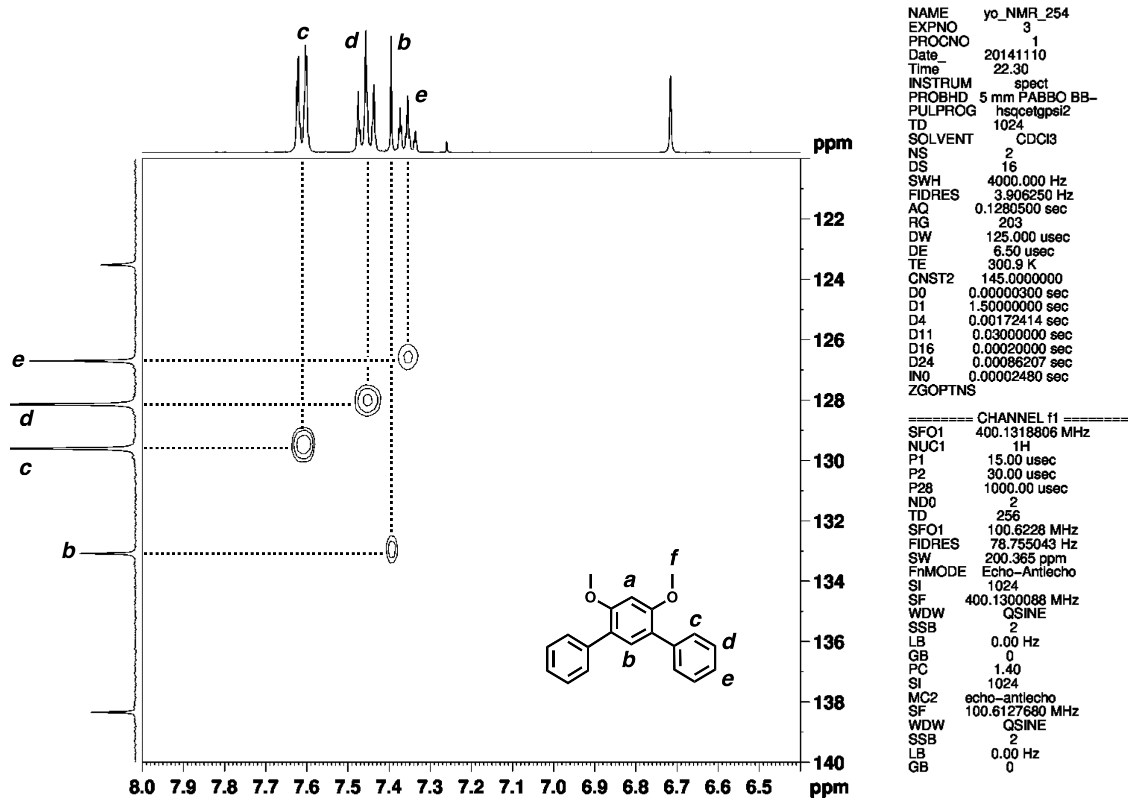

Figure S24b. HSQC spectrum (400 MHz, $CDCl_3$, r.t.) of **4b**.

Synthesis of 5b YO-12

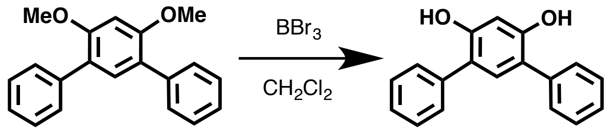

Benzene dimer **4b** (1.000 g, 3.444 mmol) and dry CH_2Cl_2 (100 mL) were added to a 200 mL glass flask. A CH_2Cl_2 solution (1.0 M) of BBr_3 (13.8 mL, 13.8 mmol) was added dropwise to the flask at 0 ºC under N_2. The reaction mixture was stirred and allowed to warm to r.t. overnight. The reaction was quenched with H_2O (50 mL). The two layers were separated and then the aqueous layer was extracted with CH_2Cl_2 (3 × 100 mL). The combined organic layers were dried over $MgSO_4$, filtrated, and concentrated under reduced pressure. The crude product was washed with acetone and hexane to afford **5b** (0.850 g, 3.24 mmol, 94% yield) as a white solid.[2]

^{1}H NMR (400 MHz, $CDCl_3$, r.t.): δ 5.34 (s, 2H), 6.65 (s, 1H), 7.16 (s, 1H), 7.37-7.40 (m, 2H), 7.45 (d, J = 4.4 Hz, 4H), 7.45 (d, J = 4.4 Hz, 4H). ^{13}C NMR (100 MHz, $CDCl_3$,

r.t.): δ 103.3 (CH), 121.5 (C_q), 127.7 (CH), 129.3 (CH), 129.4 (CH), 131.7 (CH), 136.9 (C_q), 153.3 (C_q). FT-IR (KBr, cm^{-1}): 3471, 3064, 3025, 2925, 1615, 1532, 1513, 1484, 1445, 1397, 1346, 1313, 1257, 1160, 1075, 1031, 985, 898, 841, 804, 787, 759, 700, 637, 619. GC-MS (*m*/*z*): Calcd. for $C_{20}H_{14}O_2$: 262, Found 262 $[M]^+$. E.A.: Calcd. for $C_{20}H_{14}O_2$•$0.2CH_3COCH_3$: C, 81.56; H, 5.59. Found: C, 81.56; H, 5.56.

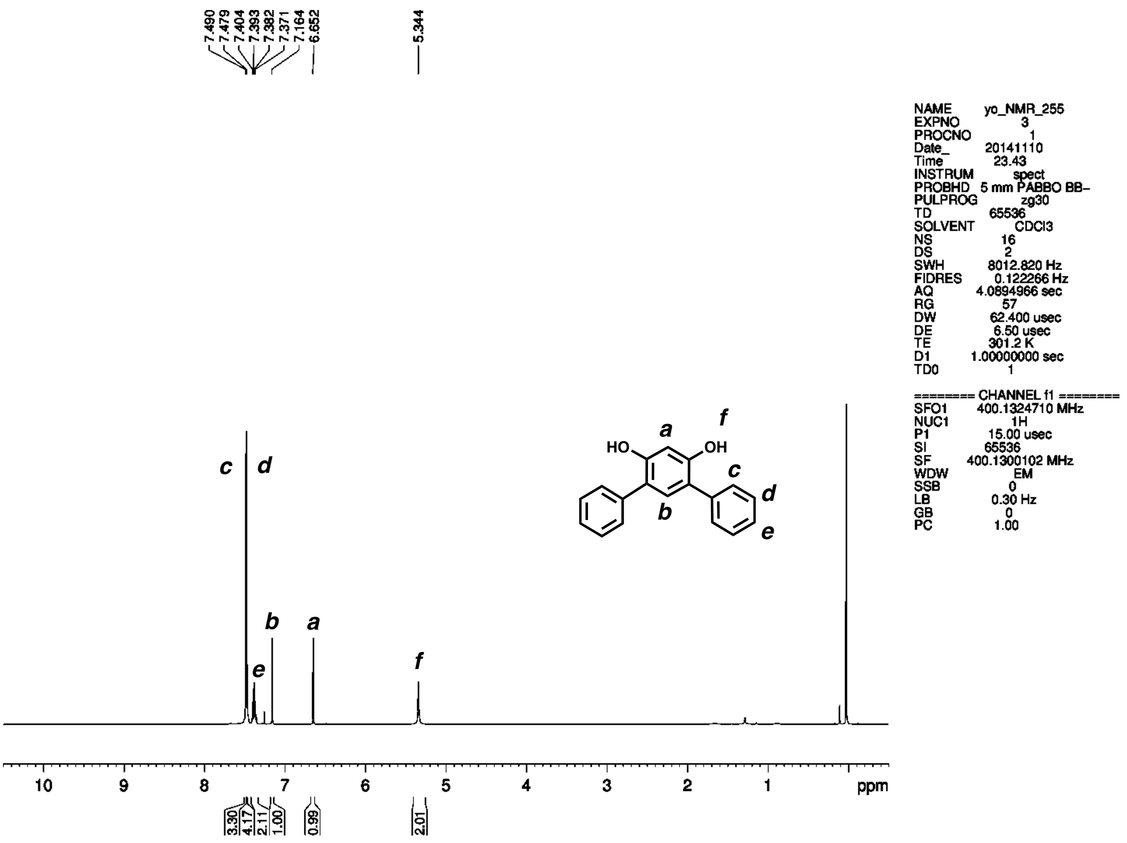

Figure S25. ^1H NMR spectrum (400 MHz, $CDCl_3$, r.t.) of **5b**.

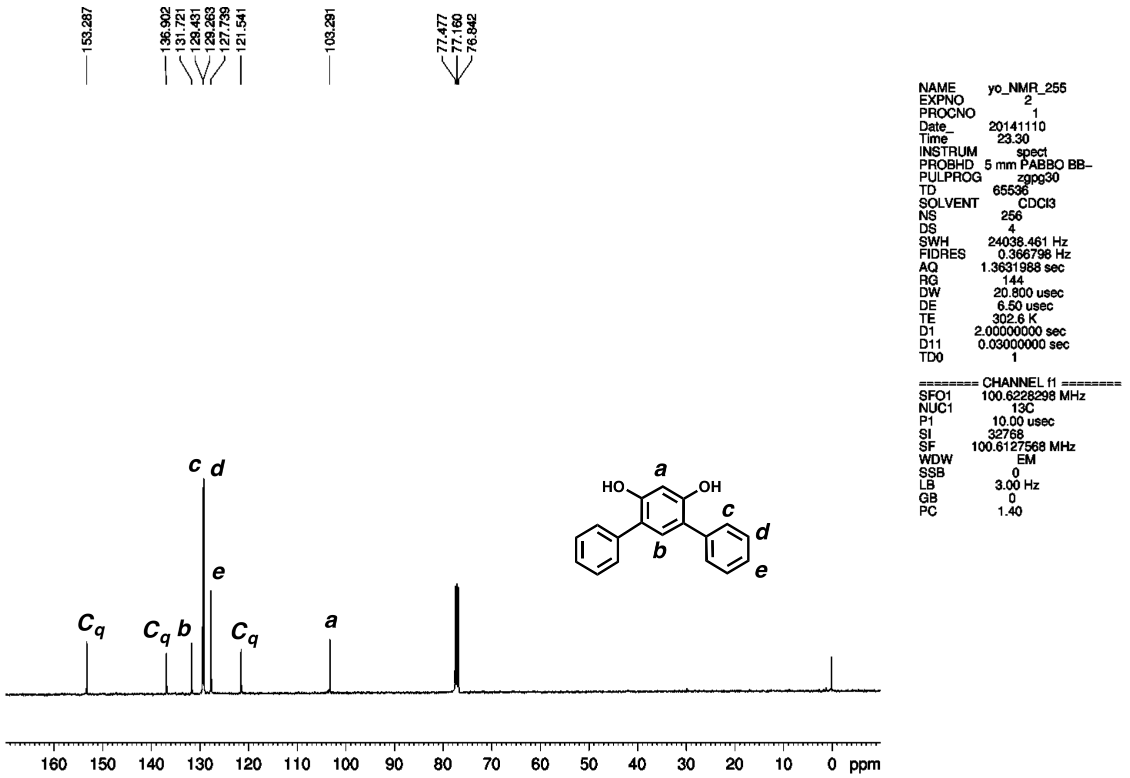

Figure S26. ^{13}C NMR spectrum (100 MHz, $CDCl_3$, r.t.) of **5b**.

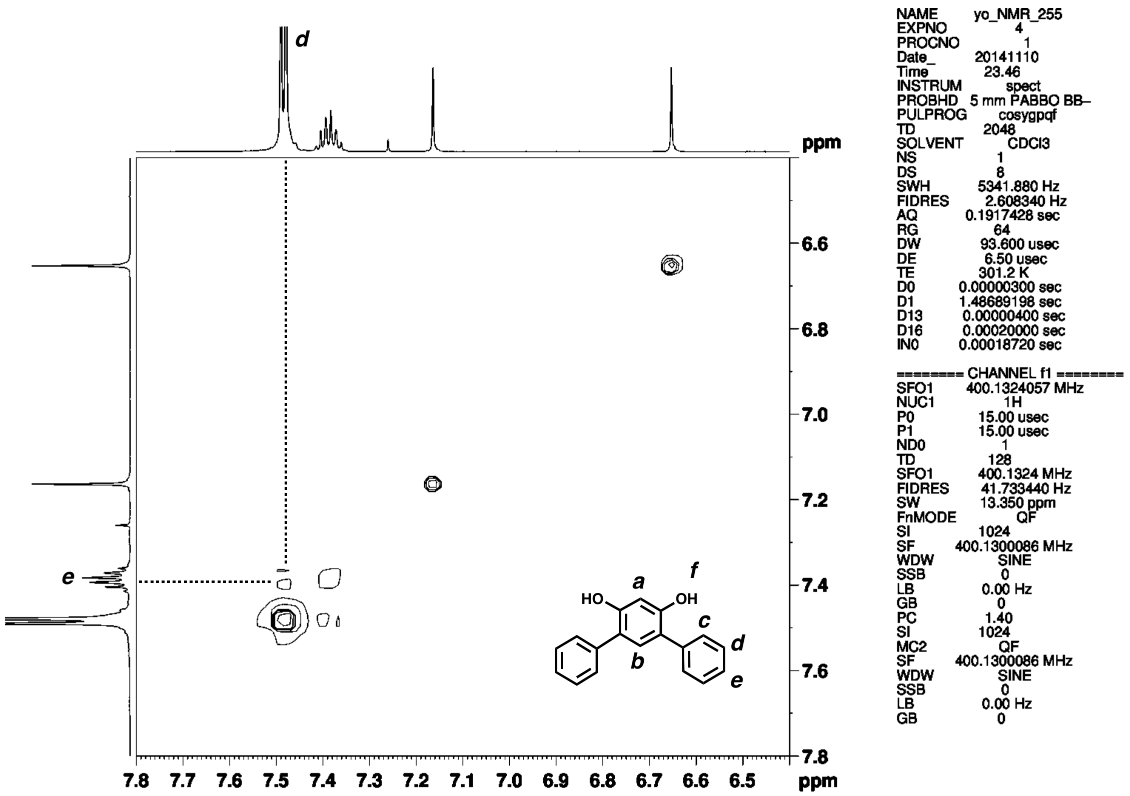

Figure S27. ^{1}H-^{1}H COSY spectrum (400 MHz, $CDCl_3$, r.t.) of **5b**.

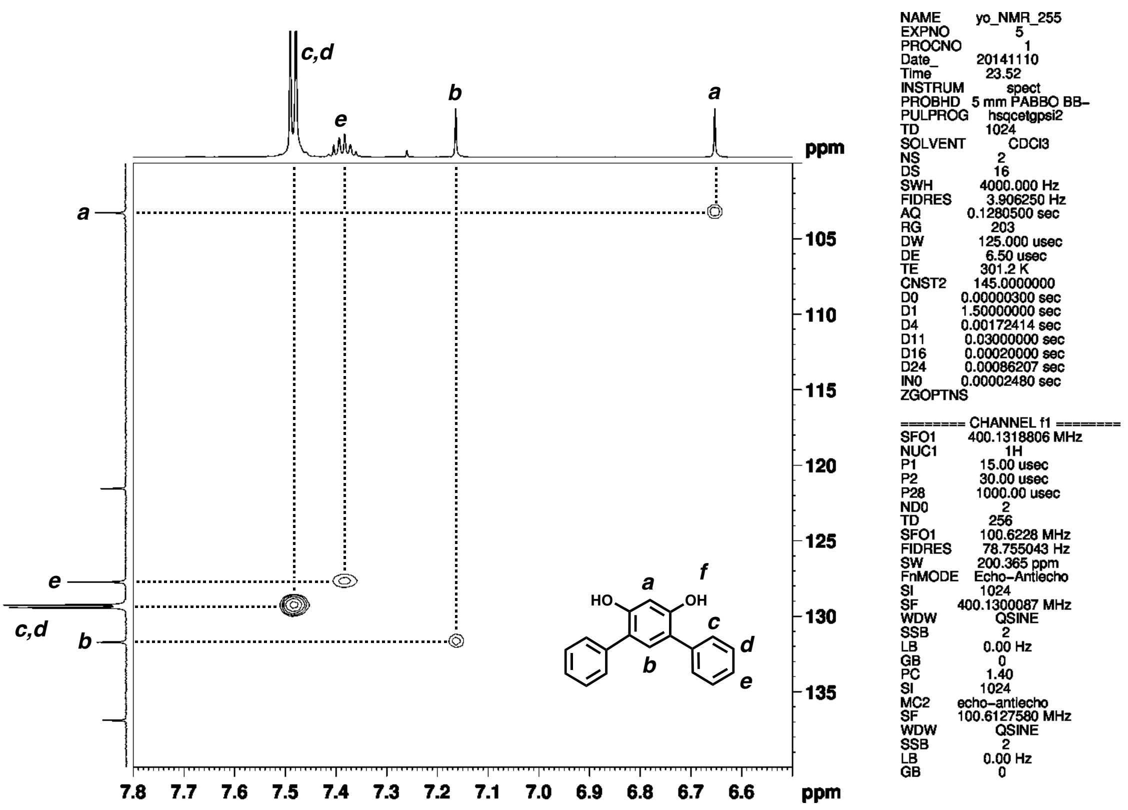

Figure S28. HSQC spectrum (400 MHz, $CDCl_3$, r.t.) of **5b**.

Synthesis of 1b   YO-13

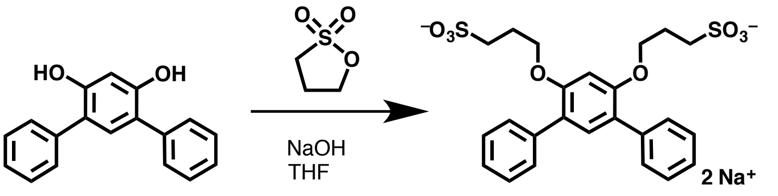

Benzene dimer **5b** (500 mg, 1.91 mmol), NaOH (458 mg, 11.4 mmol), and THF (50 mL) were added to a 50 mL glass flask. 1,3-Propanesultone (755 mg, 5.72 mmol) was added dropwise to the flask. The resultant mixture was stirred at r.t. overnight. The suspended solution was filtrated and then CH_2Cl_2 (5 mL) and hexane (50 mL) was added to the filtrate. The resultant solution was concentrated under reduced pressure. The crude product was washed with water (2 mL) and 1-propanol (20 mL) to afford **1b** (0.500 g, 0.908 mmol, 48% yield) as a white solid.

^{1}H NMR (400 MHz, CD_3OD, r.t.): δ 2.21 (dt, J = 6.8, 6.0 Hz, 4H), 2.93 (t, J = 6.8 Hz,

4H), 4.19 (t, J = 6.0 Hz, 4H), 6.83 (s, 1H), 7.21 (s, 1H), 7.24 (t, J = 7.2 Hz, 2H), 7.37 (dd, J = 8.0, 7.2 Hz, 4H), 7.50 (d, J = 8.0 Hz, 4H). ^{13}C NMR (100 MHz, CD_3OD, r.t.): δ 26.4 (CH_2), 49.6 (CH_2), 68.7 (CH_2), 100.4 (CH), 125.2 (C_q), 127.7 (CH), 128.9 (CH), 130.5 (CH), 133.4 (CH), 139.8 (C_q), 157.4 (C_q). FT-IR (KBr, cm^{-1}): 3525, 2940, 2883, 1630, 1610, 1582, 1520, 1484, 1469, 1442, 1409, 1386, 1315, 1277, 1237, 1185, 1044, 890, 816, 798, 769, 699, 656, 623. HR MS (ESI): m/z Calcd. for $C_{24}H_{24}NaO_8S_2$ 527.0805, Found 527.0807 [M–Na^+]$^-$. E.A.: Calcd. for $C_{24}H_{24}Na_2O_8S_2$•1.9H_2O: C, 49.29; H, 4.79. Found: C, 49.14; H, 4.61.

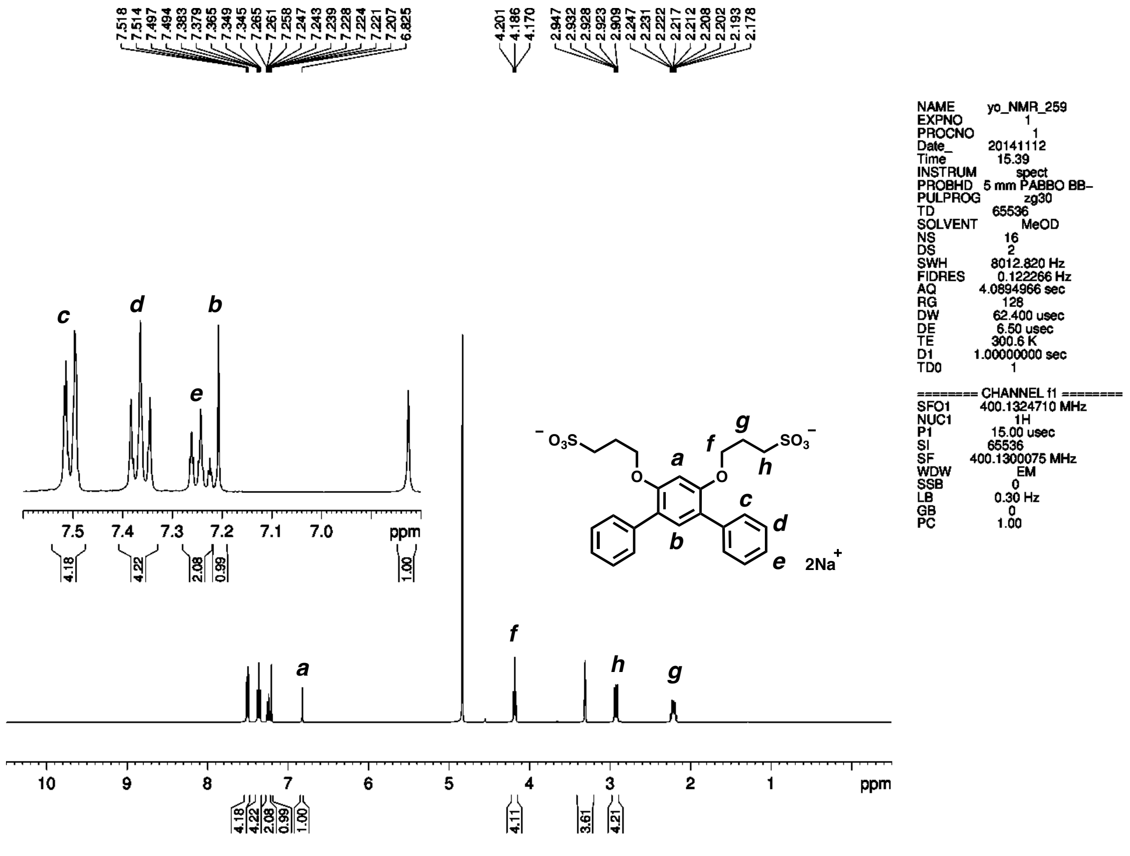

Figure S29. ^{1}H NMR spectrum (400 MHz, CD_3OD, r.t.) of **1b**.

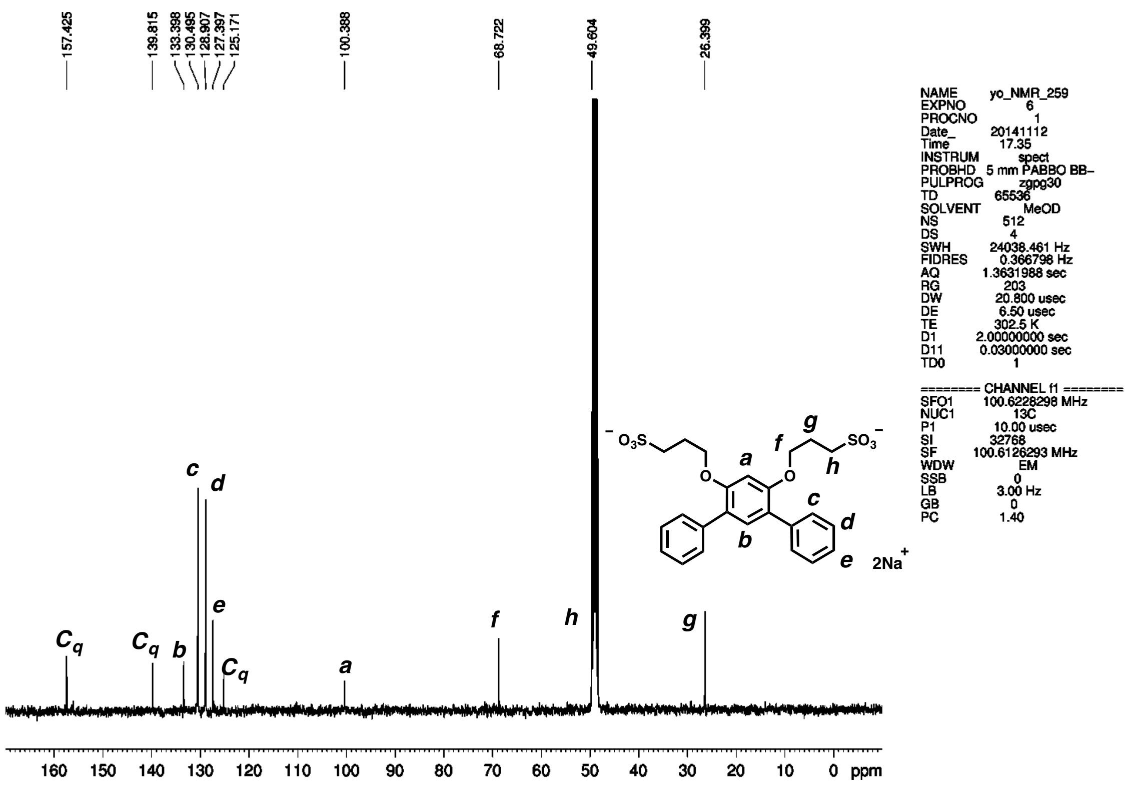

Figure S30. ^{13}C NMR spectrum (100 MHz, CD_3OD, r.t.) of **1b**.

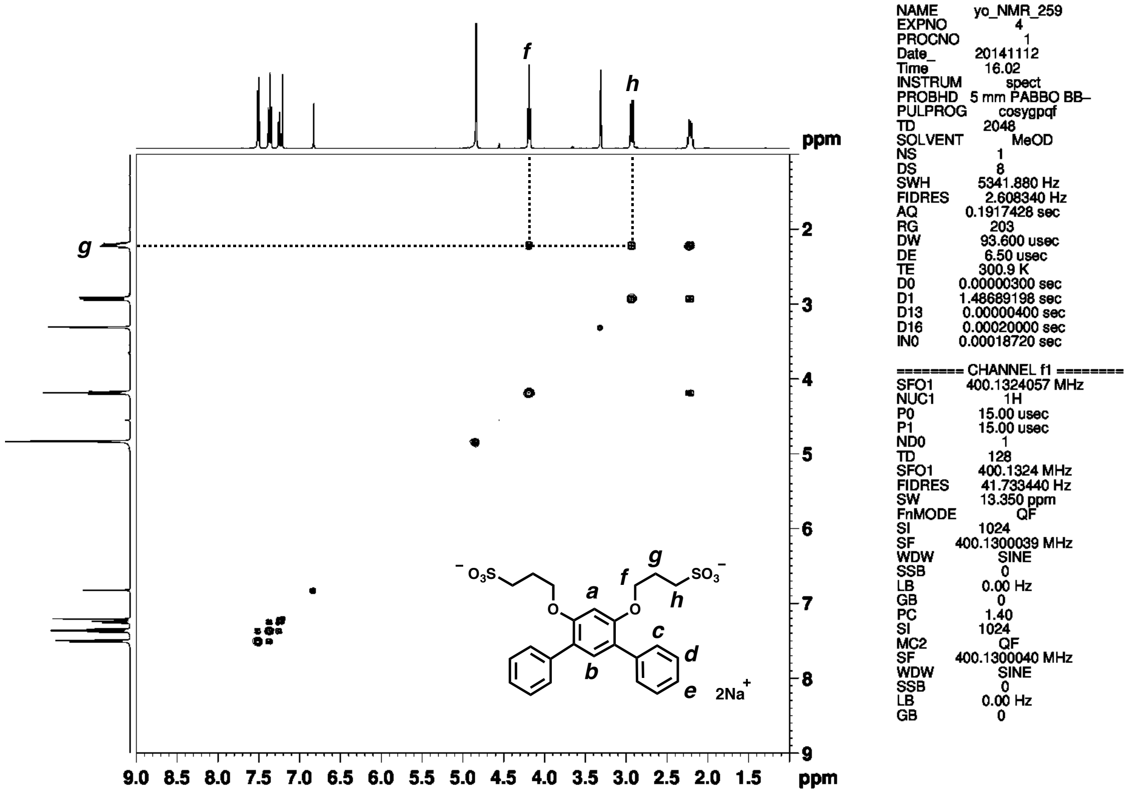

Figure S31a. ^1H-^1H COSY spectrum (400 MHz, CD_3OD, r.t.) of **1b**.

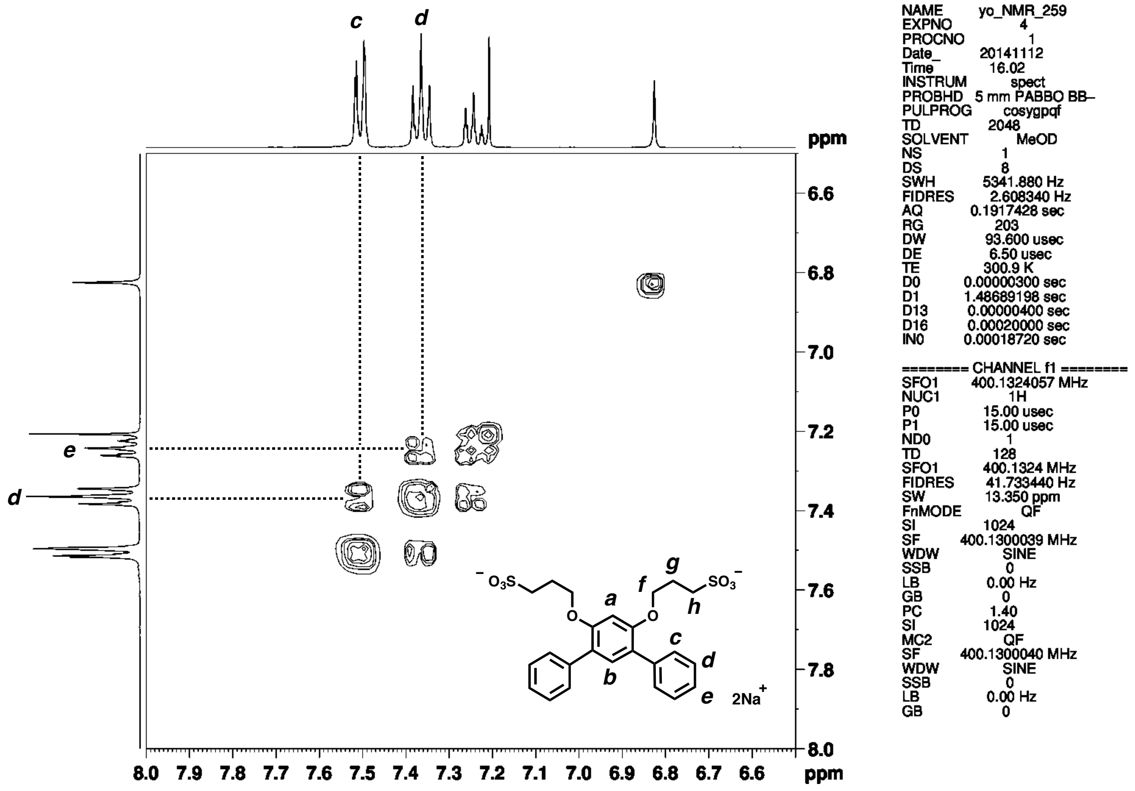

Figure S31b. ^{1}H-^{1}H COSY spectrum (400 MHz, CD_3OD, r.t.) of **1b**.

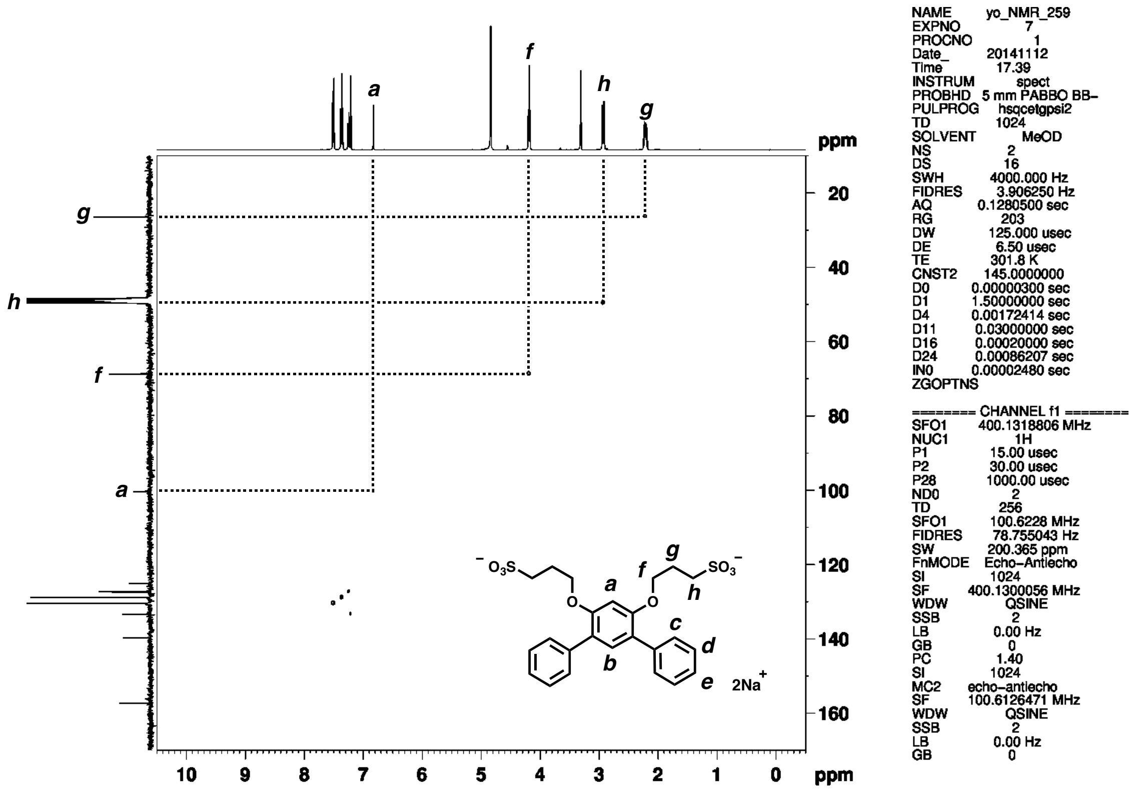

Figure S32a. HSQC spectrum (400 MHz, CD_3OD, r.t.) of **1b**.

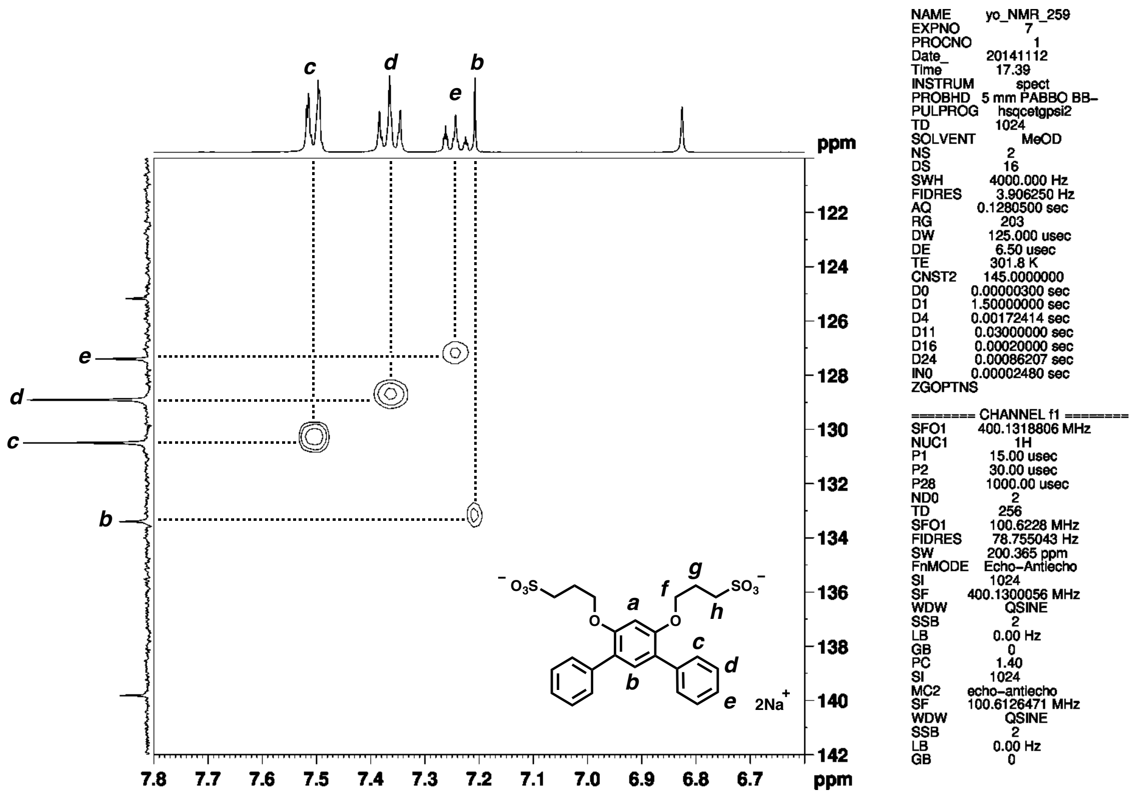

Figure S32b. HSQC spectrum (400 MHz, CD_3OD, r.t.) of **1b**.

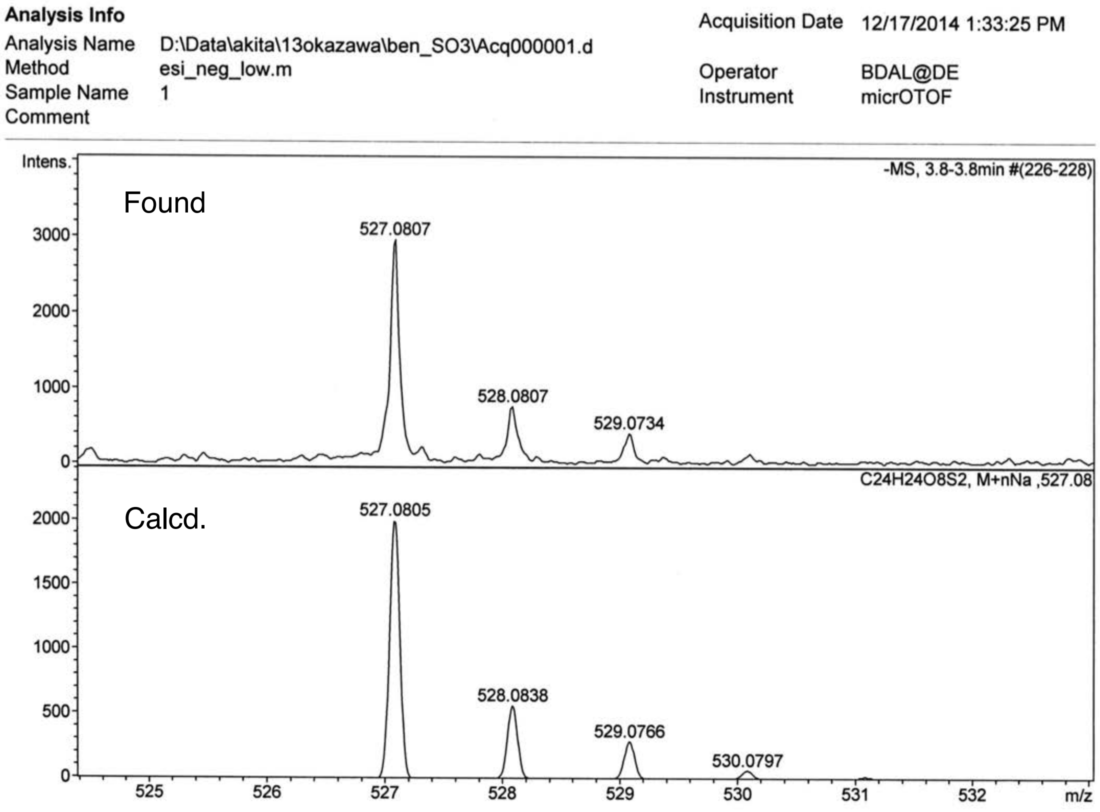

Figure S33. HR MS spectrum (ESI) of **1b**.

Self-assembly of 2b YO-20

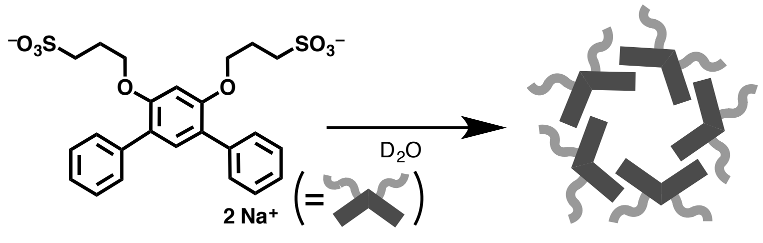

Amphiphile **1b** (0.4 mg, 0.8 μmol) and D_2O (0.4 mL) were added to a glass test tube. When the mixture was stirred at r.t. for 1 min, the formation of **2b** was confirmed by NMR, DLS, and AFM analyses.

^{1}H NMR (400 MHz, D_2O, 2.0 mM based on **1b**, r.t.): δ 2.18 (dt, J = 7.6, 6.4 Hz, 4H), 3.00 (t, J = 7.6 Hz, 4H), 4.26 (t, J = 6.4 Hz, 4H), 6.97 (s, 1H), 7.36 (s, 1H), 7.45 (t, J = 7.4 Hz, 2H), 7.54 (dd, J = 8.0, 7.4 Hz, 4H), 7.58 (d, J = 8.0 Hz, 4H). ^{13}C NMR (125 MHz, D_2O, 2.0 mM based on **1b**, r.t.): δ 26.2 (CH_2), 48.0 (CH_2), 67.7 (CH_2), 100.6 (CH), 124.4 (C_q), 127.3 (CH), 128.4 (CH), 129.5 (CH), 132.2 (CH), 137.4 (C_q), 155.5 (C_q). DOSY NMR (400 MHz, D_2O, 2.0 mM based on **1b**, 25 ºC): D = 5.01 x 10^{-10} m^2 s^{-1}.

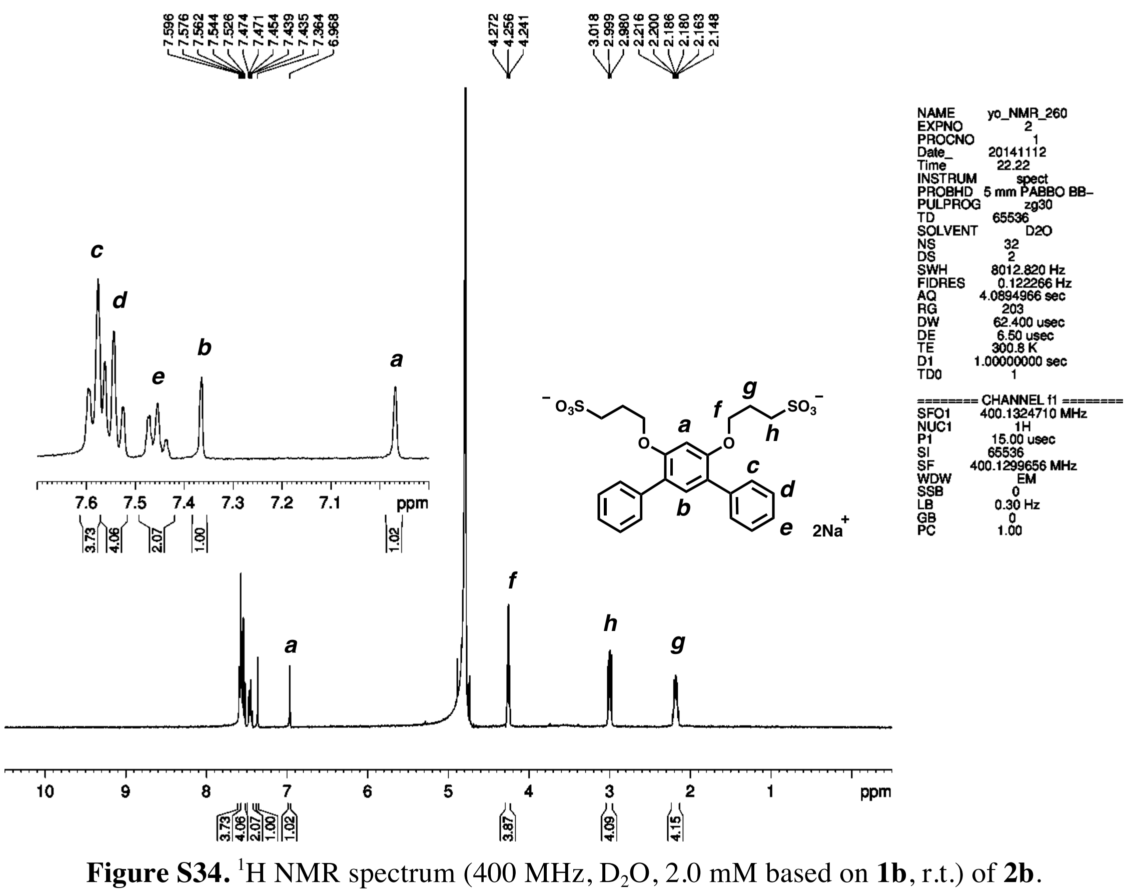

Figure S34. ^{1}H NMR spectrum (400 MHz, D_2O, 2.0 mM based on **1b**, r.t.) of **2b**.

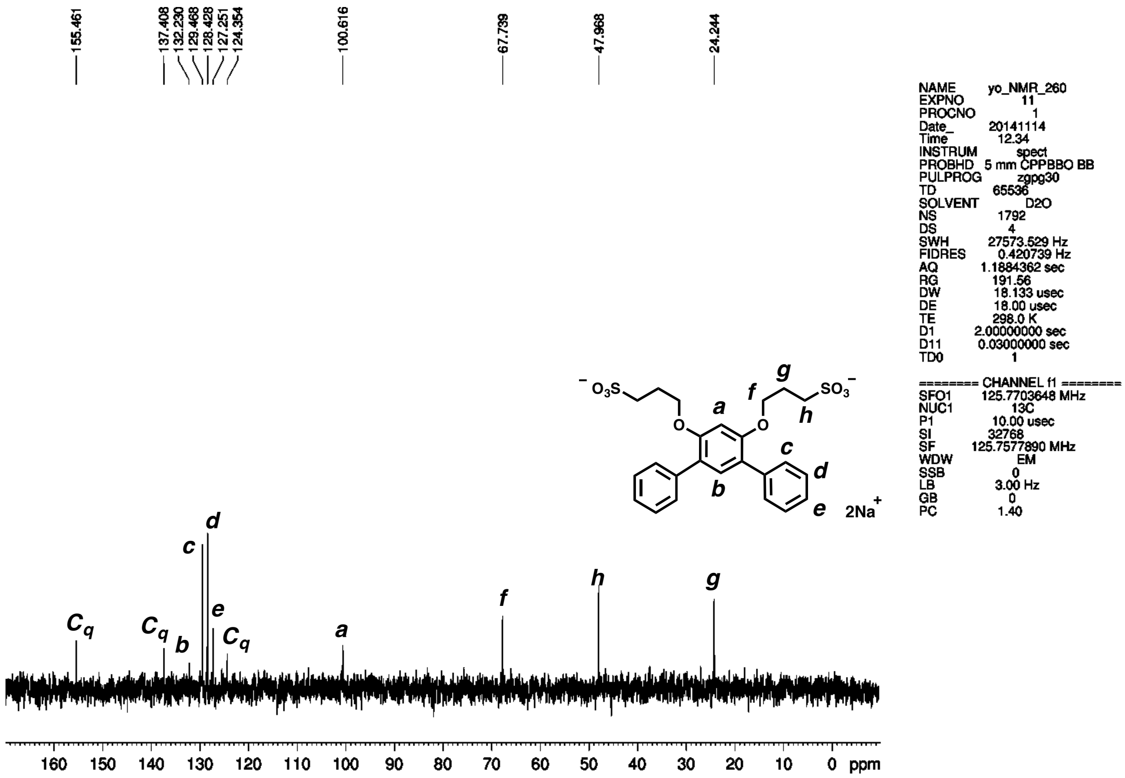

Figure S35. ^{13}C NMR spectrum (125 MHz, D_2O, 2.0 mM based on **1b**, r.t.) of **2b**.

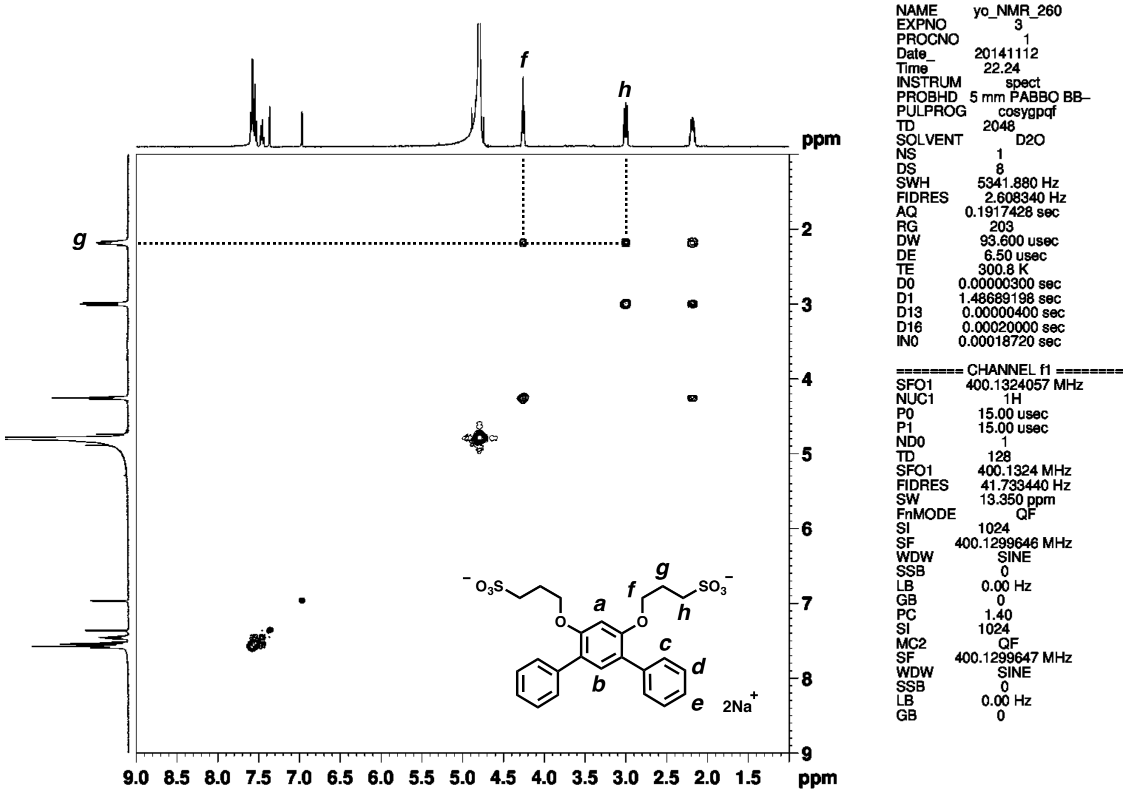

Figure S36a. ^{1}H-^{1}H COSY spectrum (400 MHz, D_2O, 2.0 mM based on **1b**, r.t.) of **2b**.

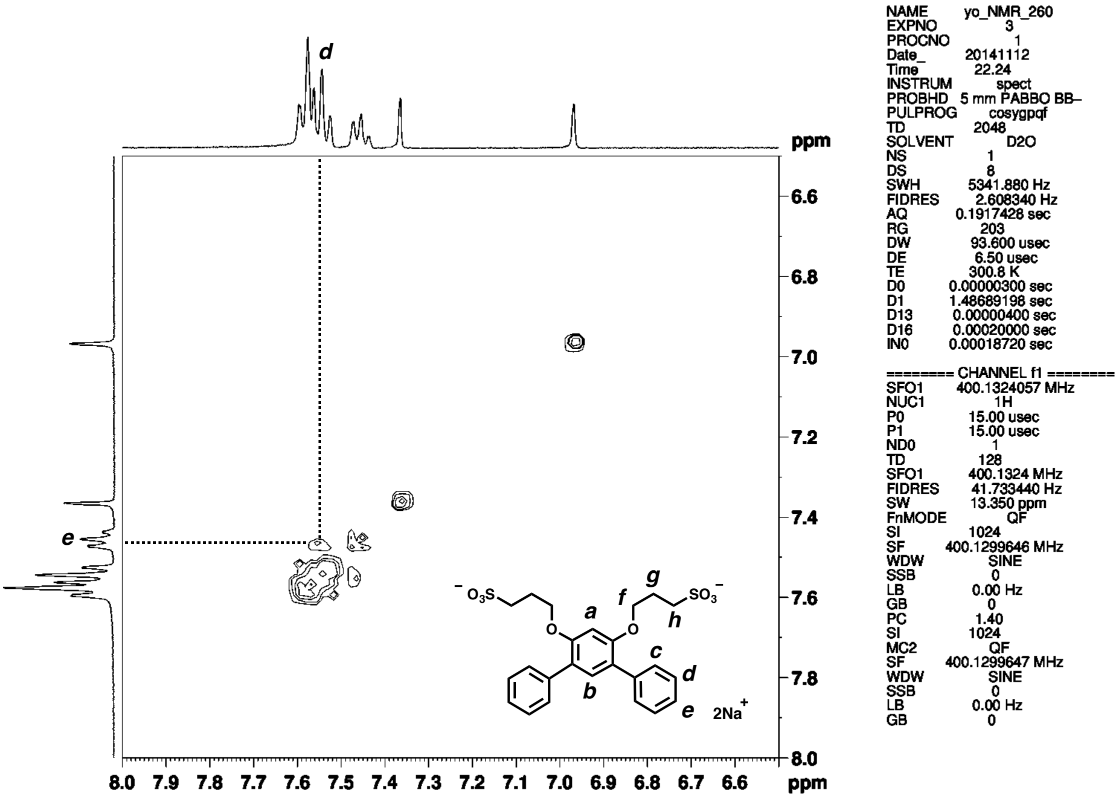

Figure S36b. ^{1}H-^{1}H COSY spectrum (400 MHz, D_2O, 2.0 mM based on **1b**, r.t.) of **2b**.

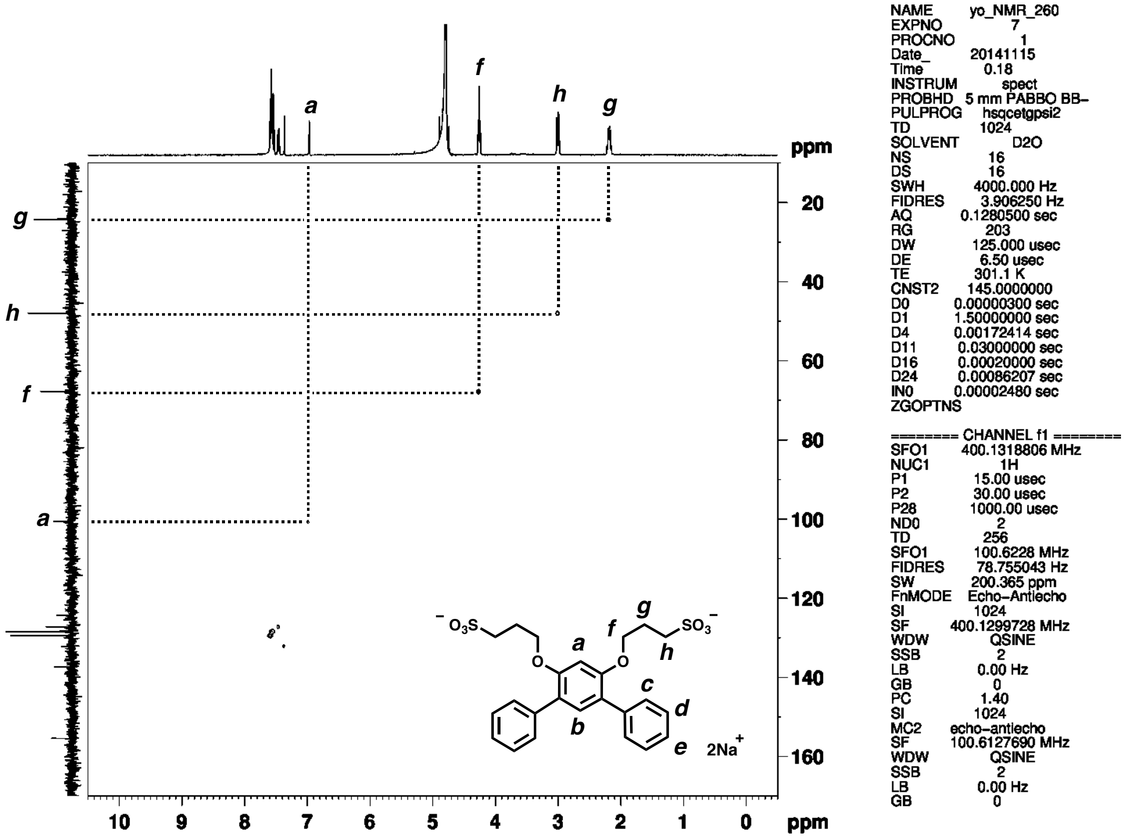

Figure S37a. HSQC spectrum (400 MHz, D_2O, 2.0 mM based on **1b**, r.t.) of **2b**.

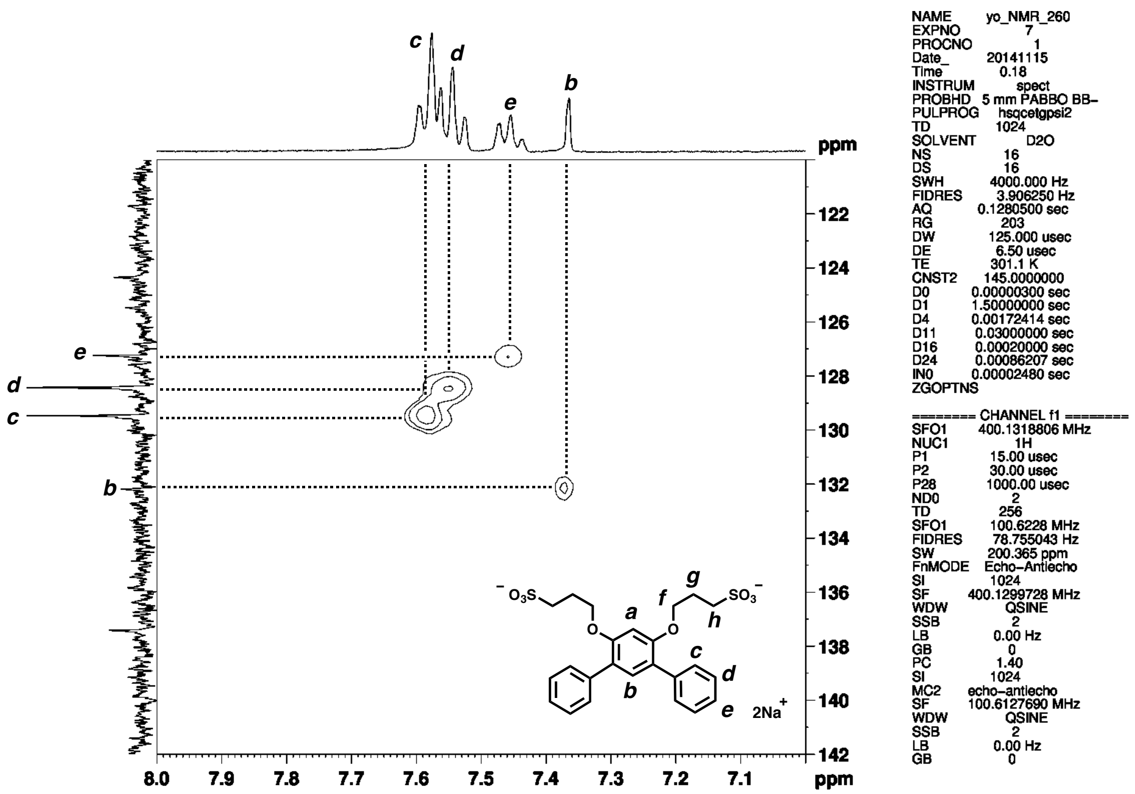

Figure S37b. HSQC spectrum (400 MHz, D_2O, 2.0 mM based on **1b**, r.t.) of **2b**.

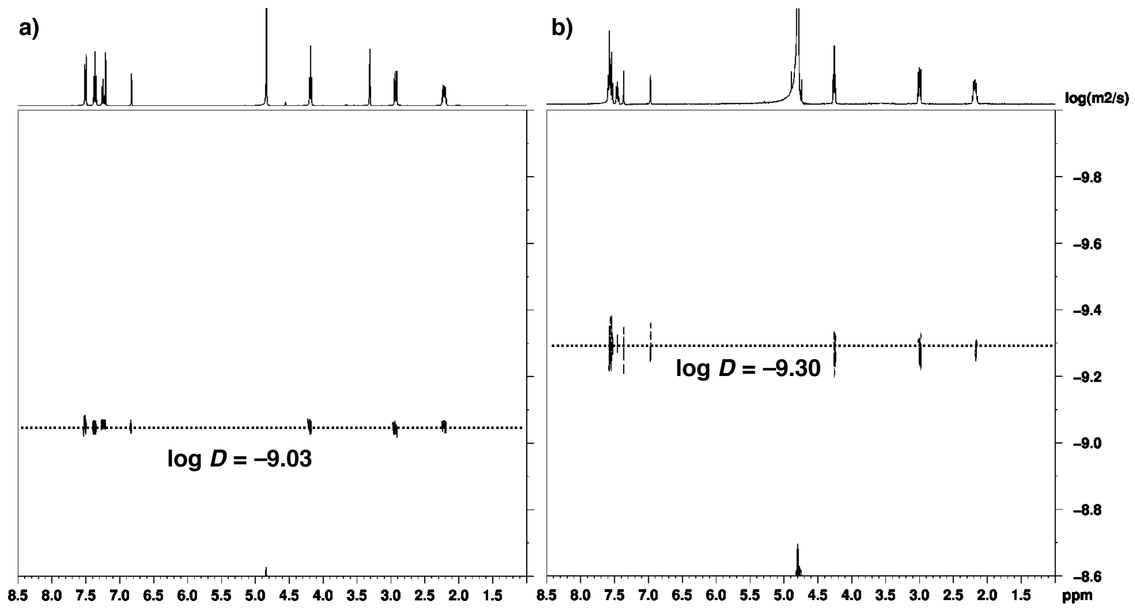

Figure S38. DOSY NMR spectra (400 MHz, 25 °C) of a) **1b** in CD_3OD and b) **2b** in D_2O (2.0 mM based on **1b**).

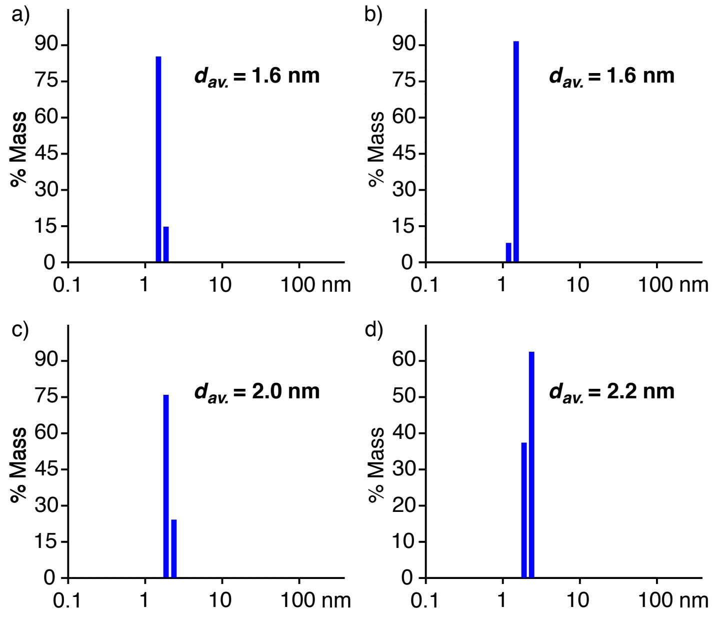

Figure S39. Concentration-dependent particle size distribution of **2a** by DLS analysis (H_2O, r.t.): a) 1.0, b) 2.0, c) 5.0, and d) 10 mM based on **1a**.

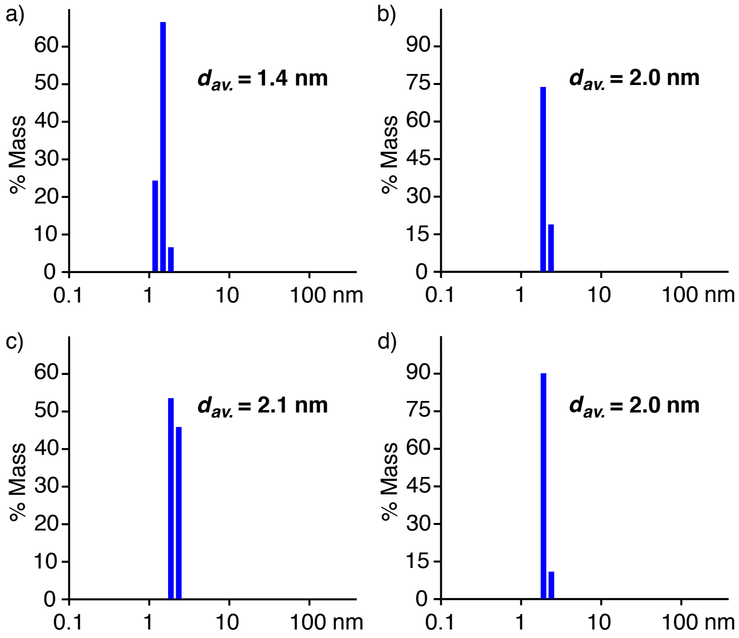

Figure S40. Concentration-dependent particle size distribution of **2b** by DLS analysis (H_2O, r.t.): a) 1.0, b) 2.0, c) 5.0, and d) 10 mM based on **1b**.

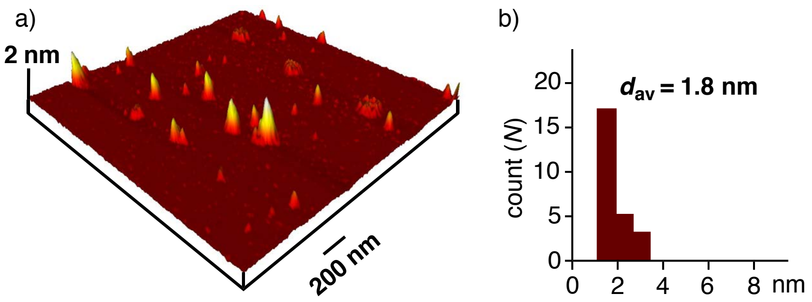

Figure S41. a) AFM image of **2b** on mica. b) the size and number (*N*) distribution.

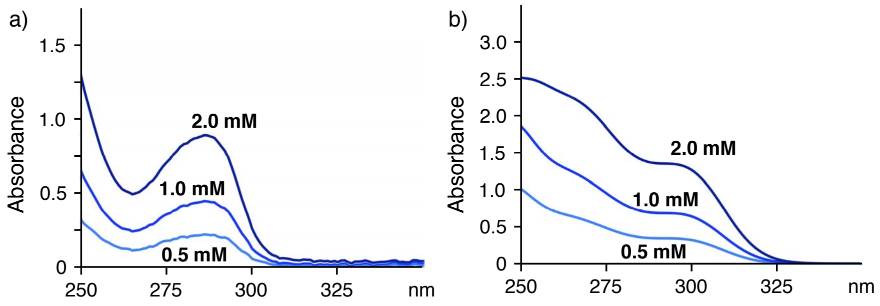

Figure S42. Concentration-dependent UV-vis spectra (H_2O, r.t.) of a) **2a** and b) **2b**.

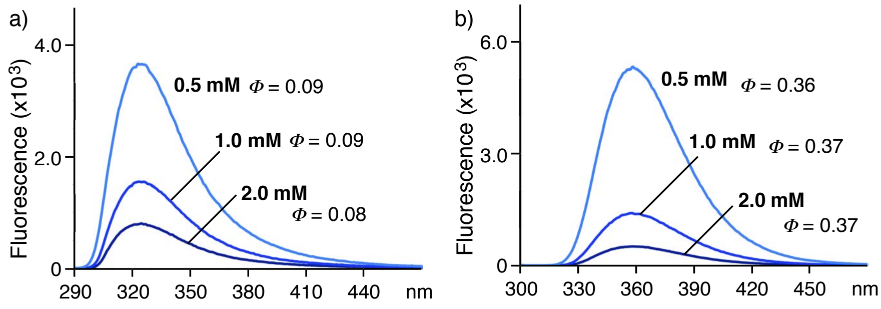

Figure S43a. Concentration-dependent fluorescence spectra (H_2O, r.t.) of a) **2a** and b) **2b**. Excitation wavelengths: λ_{ex} = 286 nm for **2a**, λ_{ex} = 299 nm for **2b**.

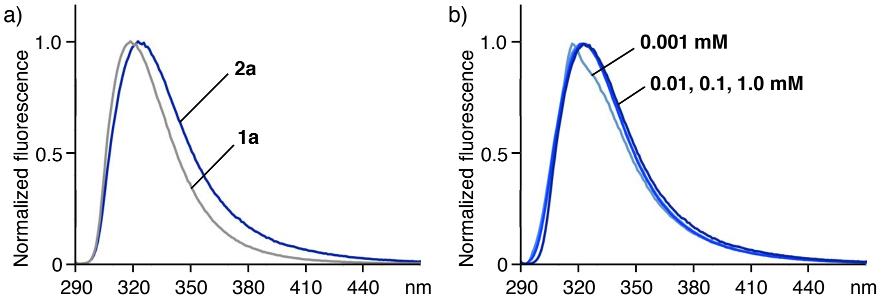

Figure S43b. a) Fluorescence spectra (λ_{ex} = 286 nm, 1.0 mM based on **1a**, r.t.) of **1a** (CH_3OH) and **2a** (H_2O). b) Concentration-dependent fluorescence spectra (λ_{ex} = 286 nm, 1.0, 0.1, 0.01, and 0.001 mM based on **1a**, r.t.) of **2a** in H_2O.

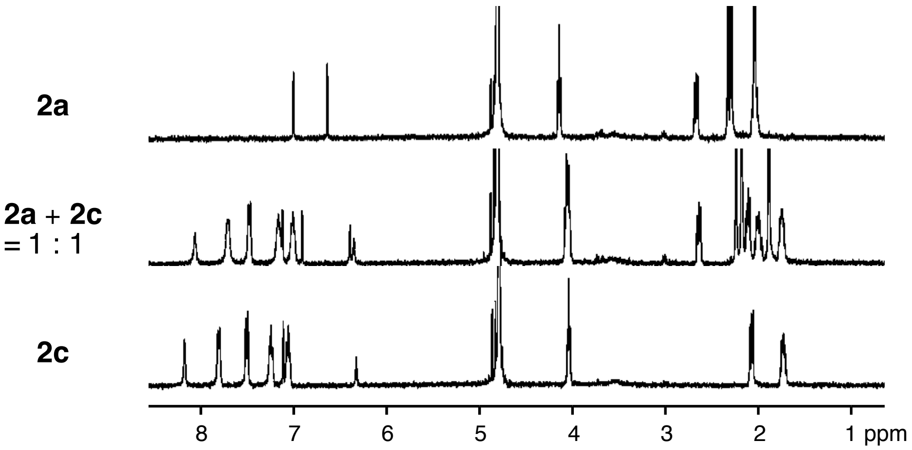

Figure S44. ^{1}H NMR spectra (400 MHz, D_2O, r.t.) of **2a**, **2c**, and **2a** + **2c** (1.0 mM based on the corresponding monomer).

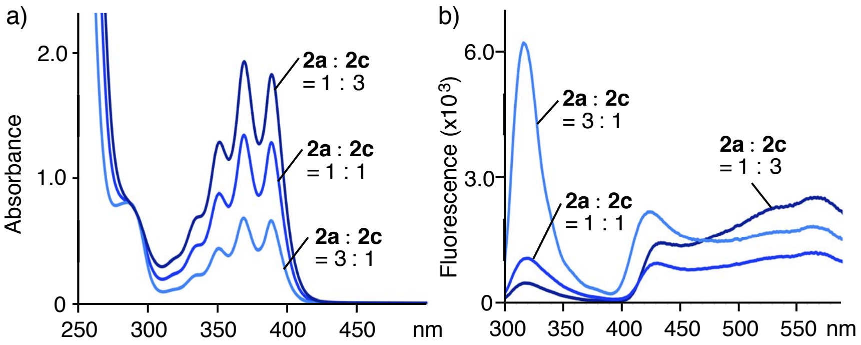

Figure S45. a) UV-vis and b) fluorescence spectra (H_2O, r.t., λ_{ex} = 286 nm) of the 1:3, 1:1, and 3:1 mixtures of **2a** and **2c** (1.0 mM based on the corresponding monomer).

Synthesis of 2a-c•(3)$_n$

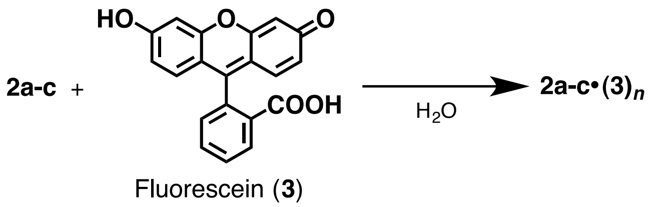

Fluorescein (**3**; 0.33 mg, 1.0 μmol) was added to a H_2O solution (2.0 mL) of **2a-c** (2.0 μmol based on **1a-c**) in a micro tube. The suspend mixture was stirred at r.t. for 1 h. After filtration, the resulting green solution including **2a-c•(3)**$_n$ was confirmed by UV-vis and fluorescence analyses.

Synthesis of 2a-c•(4)$_n$

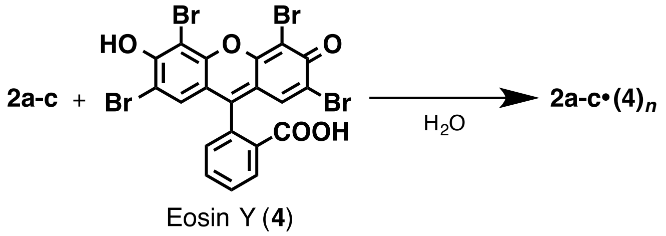

Eosin Y (**4**; 0.65 mg, 1.0 μmol) was added to a H_2O solution (2.0 mL) of **2a-c** (2.0 μmol based on **1a-c**) in a micro tube. The suspend mixture was stirred at r.t. for 1 h. After filtration, the resulting red solution including **2a-c•(4)**$_n$ was confirmed by UV-vis and fluorescence analyses.

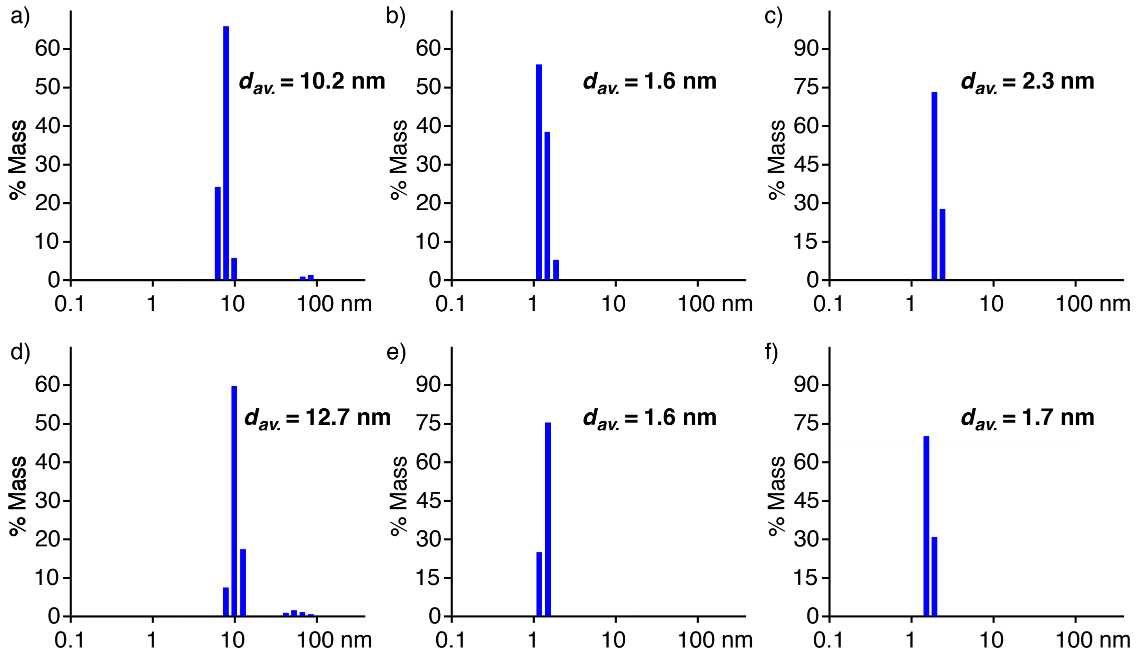

Figure S46. Particle size distribution of a) **2a•(3)**$_n$, b) **2b•(3)**$_n$, c) **2c•(3)**$_n$, d) **2a•(4)**$_n$, e) **2b•(4)**$_n$, and f) **2c•(4)**$_n$ by DLS analysis (H_2O, 1.0 mM based on the corresponding monomers, r.t.).

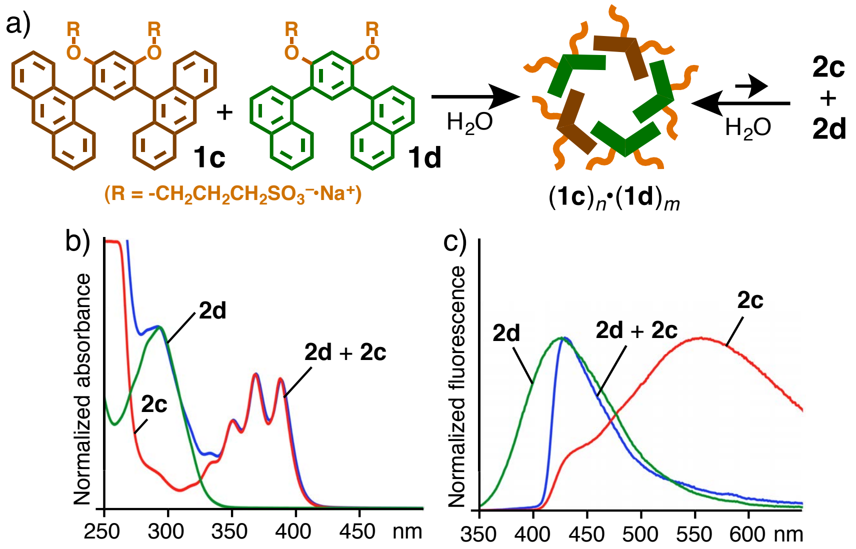

Figure S47. (a) Schematic representation of the formation of a complex mixture of nanoassemblies $(\mathbf{1c})_n$•$(\mathbf{1d})_m$ in water. (b) Normalized UV-visible and (c) fluorescence spectra (H_2O, r.t., λ_{ex} = 293 nm) of **2c**, **2d**, and **2c** + **2d** (1.0 mM based on **1c**,**d** each).

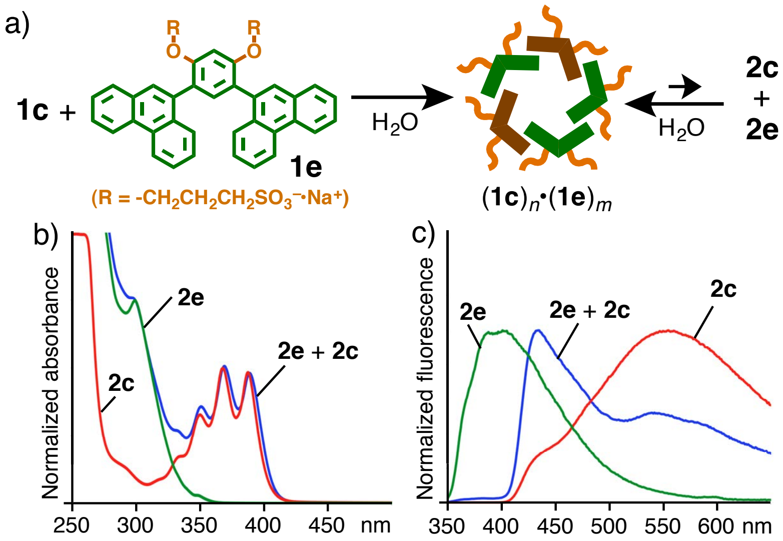

Figure S48. (a) Schematic representation of the formation of a complex mixture of nanoassemblies $(\mathbf{1c})_n$•$(\mathbf{1e})_m$ in water. (b) Normalized UV-visible and (c) fluorescence spectra (H_2O, r.t., λ_{ex} = 293 nm) of **2c**, **2e**, and **2c** + **2e** (1.0 mM based on **1c**,**e** each).

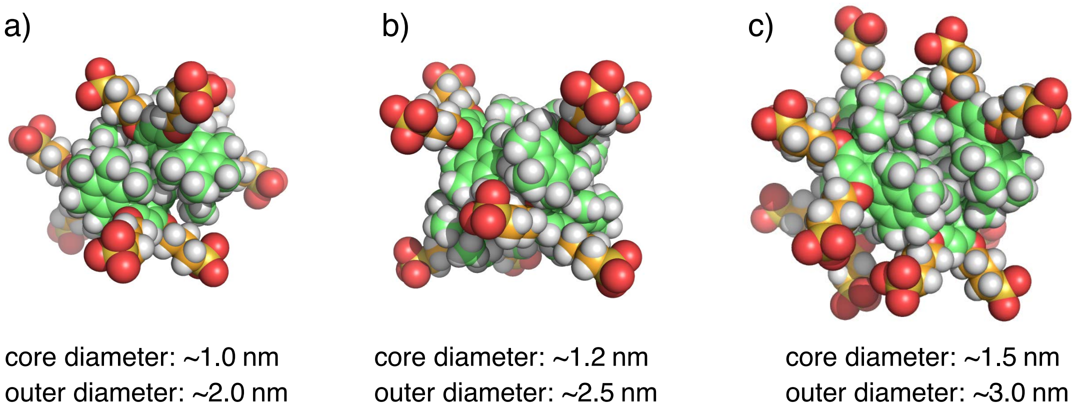

Figure S49. Optimized structures of spherical assemblies (a) (**1a**)$_4$, (b) (**1a**)$_5$, and (c) (**1a**)$_6$.

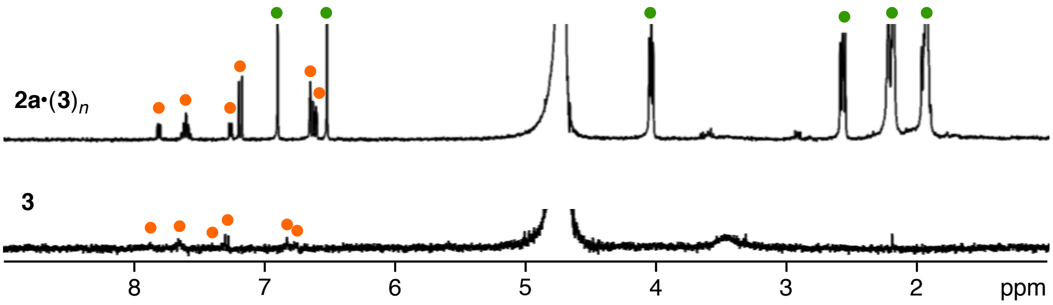

Figure S50. ^{1}H NMR spectra (400 MHz, D_2O, r.t.) of **2a•**(**3**)$_n$ (2.0 mM based on **1a**) and **3** (saturated concentration).
